# Trehalose-6-Phosphate-Mediated Toxicity Determines Essentiality of OtsB2 in *Mycobacterium tuberculosis In Vitro* and in Mice

**DOI:** 10.1371/journal.ppat.1006043

**Published:** 2016-12-09

**Authors:** Jan Korte, Marina Alber, Carolina M. Trujillo, Karl Syson, Hendrik Koliwer-Brandl, René Deenen, Karl Köhrer, Michael A. DeJesus, Travis Hartman, William R. Jacobs, Stephen Bornemann, Thomas R. Ioerger, Sabine Ehrt, Rainer Kalscheuer

**Affiliations:** 1 Institute for Pharmaceutical Biology and Biotechnology, Heinrich-Heine-University Düsseldorf, Düsseldorf, Germany; 2 Institute for Medical Microbiology and Hospital Hygiene, Heinrich-Heine-University Düsseldorf, Düsseldorf, Germany; 3 Department of Microbiology and Immunology, Weill Cornell Medical College, New York, New York, United States of America; 4 Department of Biological Chemistry, John Innes Centre, Norwich Research Park, Norwich, Norfolk, United Kingdom; 5 Biological and Medical Research Center (BMFZ), Cluster of Excellence on Plant Sciences (CEPLAS), Heinrich-Heine-University Düsseldorf, Düsseldorf, Germany; 6 Department of Computer Science, Texas A&M University, College Station, Texas, United States of America; 7 Department of Microbiology and Immunology, Howard Hughes Medical Institute, Albert Einstein College of Medicine, Bronx, New York, United States of America; National Institutes of Health, UNITED STATES

## Abstract

Trehalose biosynthesis is considered an attractive target for the development of antimicrobials against fungal, helminthic and bacterial pathogens including *Mycobacterium tuberculosis*. The most common biosynthetic route involves trehalose-6-phosphate (T6P) synthase OtsA and T6P phosphatase OtsB that generate trehalose from ADP/UDP-glucose and glucose-6-phosphate. In order to assess the drug target potential of T6P phosphatase, we generated a conditional mutant of *M*. *tuberculosis* allowing the regulated gene silencing of the T6P phosphatase gene *otsB2*. We found that *otsB2* is essential for growth of *M*. *tuberculosis in vitro* as well as for the acute infection phase in mice following aerosol infection. By contrast, *otsB2* is not essential for the chronic infection phase in mice, highlighting the substantial remodelling of trehalose metabolism during infection by *M*. *tuberculosis*. Blocking OtsB2 resulted in the accumulation of its substrate T6P, which appears to be toxic, leading to the self-poisoning of cells. Accordingly, blocking T6P production in a Δ*otsA* mutant abrogated *otsB2* essentiality. T6P accumulation elicited a global upregulation of more than 800 genes, which might result from an increase in RNA stability implied by the enhanced neutralization of toxins exhibiting ribonuclease activity. Surprisingly, overlap with the stress response caused by the accumulation of another toxic sugar phosphate molecule, maltose-1-phosphate, was minimal. A genome-wide screen for synthetic lethal interactions with *otsA* identified numerous genes, revealing additional potential drug targets synergistic with OtsB2 suitable for combination therapies that would minimize the emergence of resistance to OtsB2 inhibitors.

## Author Summary

Trehalose biosynthesis is considered an attractive target for the development of new drugs against various microbial pathogens including *Mycobacterium tuberculosis*. In this human pathogen, two partially redundant pathways mediate trehalose biosynthesis. The OtsA-OtsB2 pathway, which dominates in culture, involves trehalose-6-phosphate (T6P) synthase OtsA and T6P phosphatase OtsB2. While OtsA is dispensable, OtsB2 is strictly essential for growth of *M*. *tuberculosis*. Using conditional gene silencing, we here show that essentiality of OtsB2 is linked to accumulation of its substrate T6P, which exhibits direct or indirect toxic effects. Regulated gene expression *in vivo* revealed that OtsB2 is required to establish an acute infection of *M*. *tuberculosis* in a mouse infection model, but is surprisingly fully dispensable during the chronic infection phase. This highlights that trehalose metabolism of *M*. *tuberculosis* is substantially remodelled during infection.

## Introduction

Trehalose (α-D-glucopyranosyl-1→1-α-D-glucopyranoside) is an abundant disaccharide found in many different groups of organisms with the notable exemption of mammals. It is required for the viability and/or virulence of several fungal, helminthic and bacterial pathogens, including *Mycobacterium tuberculosis*, but not mammals, and is thus considered an attractive target for the development of antimicrobial drugs [1–9]. Among other functions in mycobacteria, trehalose provides the sugar scaffold of glycolipids such as trehalose-6,6'-dimycolate (TDM, also known as cord factor) or trehalose-6-monomycolate (TMM). These molecules fulfil crucial roles in the formation of the mycolic acid cell wall layer, either as a structural component (TDM) or as a carrier molecule (TMM) that shuttles mycolic acids from their site of biosynthesis in the cytoplasm to the periplasm, where they serve as substrates of the antigen 85 complex [10]. Based on bioinformatic analyses and enzymatic *in vitro* characterizations, it appeared that three alternative pathways for trehalose biosynthesis are potentially present in *M*. *tuberculosis*: the OtsA-OtsB2, the TreY-TreZ and the TreS pathways [11]. However, we recently demonstrated that trehalose biosynthesis is mediated in mycobacteria only by the OtsA-OtsB2 and TreY-TreZ pathways [12], whereas TreS is involved in the conversion of trehalose to alpha-glucans and therefore consumes, rather than produces, this disaccharide [13]. In the OtsA-OtsB2 pathway, the trehalose-6-phosphate (T6P) synthase OtsA catalyzes the transfer of nucleoside diphosphate-activated glucose (ADP-glucose and, to a lesser extent, UDP-glucose [14]) to glucose-6-phosphate to yield T6P with the release of ADP/UDP. Subsequently, T6P phosphatase OtsB2 dephosphorylates T6P to trehalose. The TreY-TreZ pathway releases trehalose from glucose storage polymers using first the maltooligosyltrehalose synthase TreY that converts the terminal α-1,4-glycosidic linkage at the reducing end of an α-1,4-glucan into an α-1,1-bond yielding maltooligosyltrehalose. Maltooligosyltrehalose trehalohydrolase TreZ then hydrolytically liberates trehalose. The OtsA-OtsB2 and TreY-TreZ pathways do not contribute equally to trehalose production. A Δ*otsA* (Rv3490) gene deletion mutant was significantly attenuated for *in vitro* growth in trehalose-free medium and in a mouse infection model [15], indicating that the OtsA-OtsB2 pathway is the dominant route for trehalose formation *in vitro* and *in vivo*. Surprisingly, in contrast to the genetic dispensability of *otsA* and the growth defect of the Δ*otsA* mutant, the *otsB2* gene (Rv3372) is strictly essential in *M*. *tuberculosis* because the gene could not be inactivated even in the presence of exogenous trehalose to chemically complement the biosynthetic defect [15].

The OtsA-OtsB pathway for trehalose biosynthesis (referred to as TPS-TPP or TPS1-TPS2 in other organisms) is widespread in nature and also conserved in other pathogens [7]. T6P phosphatase has been shown to contribute to virulence in the pathogenic yeasts *Candida albicans* [1–3], *Cryptococcus neoformans* [4] and *Cryptococcus gattii* [5], the filamentous fungi *Aspergillus fumigatus* [8] and *Fusarium graminearum* [6]. However, while gene deletion mutants exhibit some growth deficiencies, T6P phosphatase is not strictly required for viability in these organisms under normal *in vitro* culture conditions in contrast to *M*. *tuberculosis*. This points towards a peculiar vulnerability underlying the essential role of OtsB2 in *M*. *tuberculosis*. Thus, in this study, we generated a conditional *otsB2* mutant in *M*. *tuberculosis* allowing regulated gene silencing in order to study the basis of OtsB2 essentiality *in vitro* and to assess the viability of the mutant *in vivo* in a mouse infection model.

## Results

### OtsB2 is essential for the *in vitro* growth of *M*. *tuberculosis*

In order to test the possible essentiality of the *otsB2* gene in *M*. *tuberculosis*, we attempted gene deletion of *otsB2* in the wild type (WT) and in an isogenic merodiploid strain containing a second copy of *otsB2* provided on an integrative single-copy plasmid, using specialized transduction. Despite repeated attempts, we were unable to obtain transductants with a deleted *otsB2* gene in the haploid WT, corroborating previous observations [15]. In contrast, the endogenous *otsB2* gene could be readily deleted in the merodiploid strain, confirming that a functional copy of *otsB2* is strictly required for growth of *M*. *tuberculosis* under these conditions (S1 Fig).

**Fig 1:**
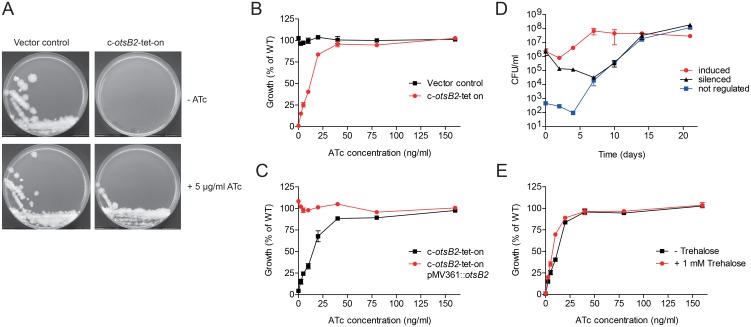
OtsB2 is essential for *in vitro* growth of *M*. *tuberculosis*. (A) The c-*otsB2*-tet-on mutant strain and a non-regulated vector control strain were grown on Middlebrook 7H10 agar with or without 5 μg/ml ATc. Plates were incubated for 21 days. For the c-*otsB2*-tet-on mutant, only growth on plates containing ATc was observed. (B) The c-*otsB2*-tet-on mutant strain was grown in liquid medium containing increasing concentrations of ATc. Strict ATc-dependency demonstrates essentiality of *otsB2* for growth in liquid medium. (C) Genetic complementation with a constitutively expressed second copy of *otsB2* abrogates ATc-dependent growth of the c-*otsB2*-tet-on mutant, showing a lack of relevant polar effects or secondary mutations. (D) Silencing of *otsB2* has a bactericidal effect. Cells of the c-*otsB2*-tet-on mutant were grown in liquid culture containing either 200 ng/ml or 0 ng/ml ATc. Culture aliquots were taken at the indicated time points, serially diluted and plated on Middlebrook 7H10 agar containing 200 ng/ml ATc to determine viable bacterial cell counts. Aliquots were plated in parallel also on 7H10 agar containing no ATc to quantify the frequency of non-regulated suppressor mutants. (E) Supplementation with 1 mM trehalose cannot compensate for the growth defect during silencing of *otsB2* in liquid culture, indicating that lack of the pathway end product is not the reason for *otsB2* essentiality. Strains in B, C and E were grown in 96-well microtiter plates for 6 days, and growth was quantified using the resazurin microplate assay. Values in B, C, and E are means of triplicates ± SEM, values in D are means of duplicates ± SEM.

In order to establish the basis of its proven genetic essentiality, we attempted conditional gene silencing of OtsB2 allowing the phenotypic characterization of partially silenced mutants. Based on a previously reported synthetic promoter cassette, which is predicated on the *Escherichia coli* Tn*10*-derived tet regulatory system and comprises a strong promoter from *M*. *smegmatis* harboring two tet operator (*tetO*) sites (P*myc1*) [16], we generated a modified promoter harboring four *tetO* sites. We reasoned that the higher number of *tetO* sites would allow more binding of tet repressor protein (TetR) to the promoter thereby increasing silencing efficacy. This promoter cassette (P*myc1*-4×*tetO*) was further engineered to include a hygromycin resistance gene for positive selection and was site-specifically inserted immediately upstream of the start codon of *otsB2* in the *M*. *tuberculosis* chromosome via specialized transduction (S2 Fig). The resulting knock-in mutant *M*. *tuberculosis* c-*otsB2*-4×*tetO* was subsequently transformed with the *E*. *coli* Tn*10 tetR* gene provided on an episomal plasmid, yielding strain *M*. *tuberculosis* c-*otsB2*-4×*tetO* pMV261::*tetR*-G. In this conditional mutant, hereafter referred to as the *M*. *tuberculosis* c-*otsB2*-tet-on mutant, expression of the target gene is repressed in the absence of the inducer anhydrotetracycline (ATc). As expected, growth of the c-*otsB2*-tet-on mutant on solid medium (Fig 1A) and in liquid culture (Fig 1B) was strictly dependent on presence of ATc, with the growth rate in liquid culture being modulated by ATc in a concentration-dependent manner. In contrast, growth of a vector control strain (i.e. the c-*otsB2*-4×*tetO* knock-in mutant transformed with an empty vector) that expresses *otsB2* constitutively, was not influenced by ATc (Fig 1B). Next, as the integrated synthetic promoter cassette might have polar effects on neighboring genes, we complemented the c-*otsB2*-tet-on mutant with a second copy of the *otsB2* gene under control of the Hsp60 promoter provided on an integrative single-copy plasmid. In this complemented *M*. *tuberculosis* c-*otsB2*-tet-on mutant, which carries a constitutively expressed merodiploid *otsB2* allele, silencing of the endogenous *otsB2* allele in the absence of ATc had no effect on growth (Fig 1C). Together these data demonstrate that OtsB2 is strictly required for viability of *M*. *tuberculosis* and rule out polar effects or inadvertent secondary mutations that could influence the silencing phenotype. Growth kinetics of the *M*. *tuberculosis* c-*otsB2*-tet-on mutant in liquid cultures showed that silencing of *otsB2* was bactericidal and resulted in a moderate killing of ca. 1.5 logs within 7 days (Fig 1D). An apparent increase of viability of silenced cells after this time point was due to the outgrowth of suppressor mutants, which might have acquired spontaneous or stress-induced mutations compromising the function of the TetR protein or, more likely, the *tet* operator sites, leading to constitutive expression of the target gene and growth of the mutants independent from ATc. Furthermore, also loss-of-function mutations in the *otsA* gene might have given rise to mutants that can grow in absence of ATc as will be discussed below.

### T6P-associated toxicity is the cause of OtsB2 essentiality

Although a Δ*otsA* mutant exhibited a growth defect *in vivo* probably due to trehalose bradytrophy [15], the *otsA* gene is in principle dispensable for *in vitro* growth of *M*. *tuberculosis*, indicating that the alternative TreX-TreY-TreZ pathway can produce sufficient amounts of trehalose to maintain viability under this condition. Therefore, we hypothesized that the essentiality of *otsB2* is not due to the limited formation of trehalose. To test this, we performed silencing experiments with the *M*. *tuberculosis* c-*otsB2*-tet-on mutant in the presence of 1 mM trehalose to chemically compensate for the possible biosynthetic defect. As expected, trehalose supplementation could not prevent growth inhibition upon *otsB2* silencing (Fig 1E), proving that lack of the pathway end product is not the basis of OtsB2 essentiality.

**Fig 2:**
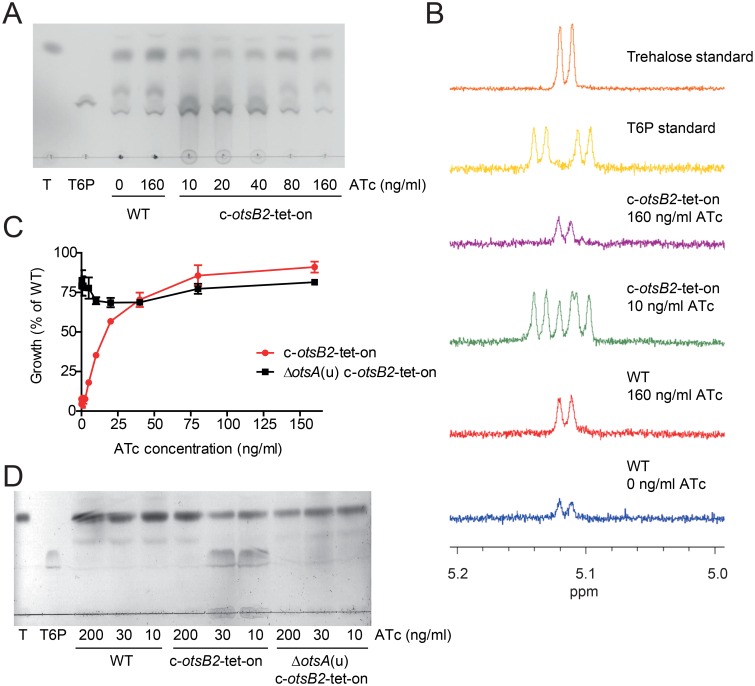
Silencing of *otsB2* leads to T6P accumulation in *M*. *tuberculosis*. (A) Cells of *M*. *tuberculosis* wild-type and the c-*otsB2*-tet-on mutant were grown in Middlebrook 7H9 liquid medium at different ATc concentrations for 6 days. Hot water extracts obtained from 7.5×10^6^ cells were analyzed by thin-layer chromatography, demonstrating the gradual accumulation of a substance co-migrating with an authentic T6P standard. Given that the volume of the cytosol of *M*. *tuberculosis* cells is 0.21 μm^3^ on average [49], the cytosolic T6P concentration in stressed cells can be estimated to be in the range from 5–10 mM. (B) ^1^H-NMR spectroscopy confirms the presence of T6P in hot water extracts of partially silenced cells of the c-*otsB2*-tet-on mutant, whereas no T6P was detectable in extracts of fully induced cells of the c-*otsB2*-tet-on mutant or *M*. *tuberculosis* wild-type. (C) Silencing of *otsB2* in a Δ*otsA*(u) mutant does not result in a growth defect. Cells were grown in 96-well microtiter plates for 6 days, and growth was quantified using the resazurin microplate assay. Values are means of triplicates ± SEM. (D) Silencing of *otsB2* in a Δ*otsA*(u) mutant does not result in T6P accumulation. Cells were grown in Middlebrook 7H9 liquid medium at different ATc concentrations for 6 days. Hot water extracts obtained from 7.5×10^6^ cells were analyzed by thin-layer chromatography.

We have recently described the essential maltosyltransferase GlgE, which is the key enzyme in a novel alpha-glucan pathway in *M*. *tuberculosis* [17, 18]. Inactivation of GlgE causes intracellular accumulation of its phosphosugar substrate, maltose-1-phosphate (M1P), which is associated with direct or indirect toxicity leading to killing of bacterial cells *in vitro* and *in vivo* by eliciting pleiotropic stress responses [13, 19]. We speculated that a similar scenario might occur during *otsB2* silencing with hyper-accumulation of the OtsB2 substrate T6P, which might be associated with toxic effects. Therefore, cell extracts from fully induced and gradually silenced cells of the *M*. *tuberculosis* c-*otsB2*-tet-on mutant were prepared and analyzed by thin-layer chromatography. While no difference compared to WT cells was observed in the fully induced conditional mutant, gradual silencing of the *otsB2* gene at low ATc concentrations resulted in the accumulation of increasing amounts of a compound that co-migrated with an authentic T6P standard (Fig 2A). ^1^H-NMR spectroscopic analyses of the same extracts confirmed the high abundance of T6P in the partially silenced conditional mutant, whereas this phosphosugar was not detectable in extracts of the fully induced conditional mutant or the WT (Fig 2B).

We hypothesized that similar to the reported toxic effects of M1P accumulation [13], intracellular T6P accumulation is toxic for *M*. *tuberculosis*. Consequently, the prevention of phosphosugar formation should avoid poisoning and abolish the essentiality of OtsB2. To block T6P synthesis, we first inactivated the T6P synthase gene *otsA* in *M*. *tuberculosis* and generated an unmarked gene deletion mutant (Δ*otsA*(u)) (S3 Fig). We then attempted deletion of the *otsB2* gene in WT and in the Δ*otsA*(u) mutant. As observed before, we were unable to inactivate *otsB2* in the WT. In contrast, transductants with a deleted *otsB2* gene were readily obtained in the Δ*otsA*(u) genetic background (S1 Fig). We also generated a conditional *otsB2* mutant in the Δ*otsA*(u) background (S2 Fig). Silencing of *otsB2* in this *M*. *tuberculosis* Δ*otsA*(u) c-*otsB2*-tet-on mutant did not lead to growth impairment in liquid culture (Fig 2C) or to the accumulation of T6P as determined by thin-layer chromatography (Fig 2D). These results unambiguously show that the essentiality of OtsB2 is dependent on the synthesis of T6P via OtsA and that the growth inhibitory consequence of *otsB2* inactivation relies on direct or indirect toxic effects associated with T6P accumulation. Exogenous T6P did not cause any growth inhibition of *M*. *tuberculosis* WT at concentrations up to 1 mM nor did it aggravate growth inhibition of the c-*otsB2*-tet-on mutant at low ATc concentrations (S4 Fig). However, this cannot be interpreted as lack of evidence for direct toxicity of T6P since this charged phosphorsugar probably cannot penetrate the cells so that only endogenously formed T6P is toxic.

### Insights into the T6P-induced stress response

**Fig 3:**
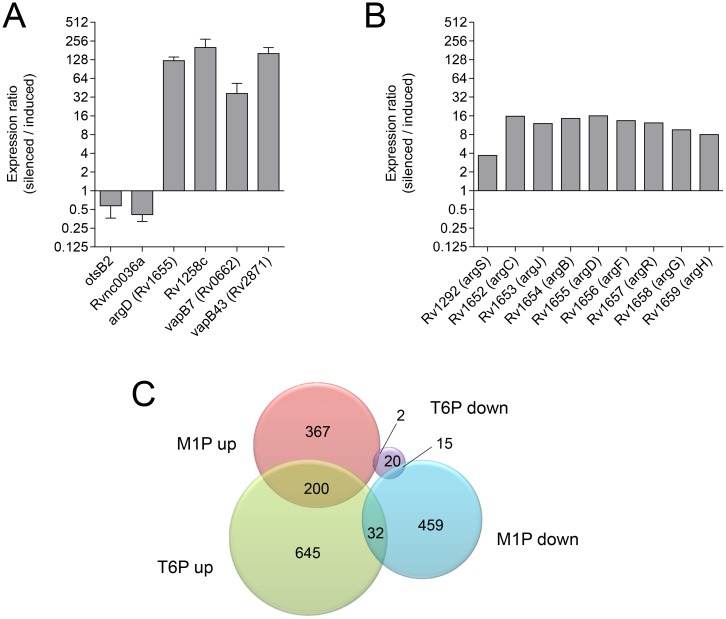
Genome-wide characterization of the T6P-elicited stress response profile. (A) Quantitative real time PCR analyses of selected transcripts to corroborate RNAseq results. qRT-PCR data were normalized to 16S rRNA, and the expression ratios of partially silenced cells of the *M*. *tuberculosis* c-*otsB2*-tet-on mutant compared to fully induced cells are reported as means of triplicates ± SEM. (B) Upregulation of arginine biosynthesis genes in T6P stressed cells of the partially silenced c-*otsB2*-tet-on mutant according to RNAseq data. (C) Diagram showing the overlap of the T6P stress response with the M1P stress response. Microarray data for M1P stress have been reported previously [13] and were obtained through NCBI Gene Expression Omnibus (GEO data set GSE18575). Only genes with a differential regulation of ≥ 2 are included.

Similar to what we have observed in *M*. *tuberculosis*, deletion of the T6P phosphatase gene results in T6P accumulation in various yeasts [1–4, 20] and filamentous fungi [6, 8]. In contrast to *M*. *tuberculosis*, however, these fungal mutants could tolerate this phosphosugar relatively well and remained viable, albeit with some growth deficiencies. Thus, *M*. *tuberculosis* exhibits unprecedented sensitivity toward T6P toxicity. In order to gain insights into the basis of toxicity, we analyzed the T6P-induced stress response by comparing the transcriptome profiles of fully induced and partially silenced cells of the *M*. *tuberculosis* c-*otsB2*-tet-on mutant employing RNAseq. In total, 877 genes were found to be significantly upregulated while only 37 genes were downregulated (≥ 2-fold with p < 0.01) in T6P stressed cells (S1 Dataset). This unexpectedly biased global shift in gene expression levels suggests that differential RNA stability rather than transcriptional regulation might primarily drive the response. In fact, antitoxins (VapB7, VapB43), which neutralize cognate toxins exhibiting RNase activity (VapC7, VapC43), were among the strongest upregulated genes (36- and 160-fold induction, respectively, in T6P-stressed cells according to qRT-PCR analysis) (Fig 3A, Table 1), implying a reduction in RNase activity and an associated increase in RNA half-life in T6P-stressed cells.

**Table 1:** List of the ten most differentially up- and downregulated genes in T6P-stressed *M*. *tuberculosis* cells. Cells of the conditional *M*. *tuberculosis* c-*otsB2*-tet-on mutant were either induced in the presence of 200 ng/ml (100% growth relative to WT) or partially silenced in the presence of 30 ng/ml ATc (ca. 30% residual growth relative to WT).

	Rv number	Gene	Fold change (silenced / induced)	Transformed p-value
Upregulated	Rv1258c	Rv1258c	27.12	0.00010
	Rv0662c	*vapB7*	23.29	0.00005
	Rv1257c	Rv1257c	19.22	0.00025
	Rv1655	*argD*	15.99	0.00018
	Rv2164c	Rv2164c	15.92	0.00041
	Rv1652	*argC*	15.72	0.00006
	Rv2165c	Rv2165c	15.54	0.00230
	Rv1654	*argB*	14.48	0.00017
	Rv1656	*argF*	13.38	0.00001
	Rv1987	Rv1987	12.46	0.00009
Downregulated	Rv2628	Rv2628	0.20	0.00066
	Rv2989	Rv2989	0.20	0.00004
	Rv2990c	Rv2990c	0.17	0.00162
	RVnc0035	MTS1082	0.16	0.00106
	Rv2988c	*leuC*	0.15	0.00009
	RVnc0036a	MTS2823	0.14	0.00021
	Rv2987c	*leuD*	0.14	0.00036
	Rv2056c	*rpsN2*	0.14	0.00030
	Rv0280	PPE3	0.09	0.00182
	RVnc0036	MTS1338	0.05	0.00008

The most upregulated gene Rv1258c (>200-fold induction in T6P-stressed cells according to qRT-PCR analysis) (Fig 3A, Table 1) encodes a putative efflux pump potentially involved in antibiotic resistance. However, T6P-stressed cells showed unaltered susceptibility to the first-line antibiotics rifampicin, ethambutol and isoniazid (S5 Fig), demonstrating that OtsB2 inactivation does not provoke general intrinsic drug resistance. Furthermore, genes involved in arginine biosynthesis were highly upregulated in response to T6P stress (Fig 3B, Table 1). A similar response has also been observed in M1P-stressed *M*. *tuberculosis* cells [13]. suggesting that arginine might play a role in counteracting stress elicited by sugarphosphates. However, arginine supplementation (1 mM) didn´t rescue growth of the c-*otsB2*-tet-on mutant at low ATc concentrations, providing no support for arginine having a direct protective effect during T6P stress (S6 Fig). Additionally, upregulation of several DNA damage-inducible genes including those belonging to the SOS regulon [21] were indicative of direct or indirect DNA damage caused by T6P stress (S7 Fig), again reminiscent of the M1P stress response [13]. Apart from these two common signatures, however, there was unexpectedly little overlap with the transcriptome profile elicited by M1P poisoning [13], indicating that these two phosphosugars induce remarkably different stress responses (Fig 3C). In addition to tRNAs, several non-coding RNAs were among the most abundant transcripts present in *M*. *tuberculosis* (S1 Table, S1 Dataset). One of these highly expressed non-coding RNAs, Rvnc0036a (= MTS2823), was one of the few genes significantly downregulated in response to T6P stress (Fig 3A, Table 1). While its expression appears to correlate with different stresses, its function is unknown [22]. However, the ultrahigh abundance in fully induced cells suggests an important physiological role under the tested culture conditions, and its depletion in partially silenced cells might contribute to the inability to tolerate the toxic effects of T6P accumulation.

### OtsB2 is essential for *M*. *tuberculosis* to establish an acute infection in mice

T6P is formed from intermediates of primary sugar metabolism (i.e. glucose-6-phosphate and ADP/UDP-glucose). However, *M*. *tuberculosis* primarily uses host lipids as sources of carbon and energy during infection given the limited carbohydrate availability [23]. It was therefore questionable whether sufficient amounts of T6P are produced by *M*. *tuberculosis in vivo* in a carbohydrate-poor environment to reach toxic intracellular concentrations. To address this question, silencing experiments in a mouse infection model were necessary.

With the genetic system described here, regulated gene expression relies on the presence of the episomal TetR expression plasmid. Loss of this plasmid would abrogate regulation and result in constitutive expression of the target gene. For stabilization of the episomal TetR expression plasmid in the absence of antibiotics *in vivo*, we employed auxotrophy complementation of the *M*. *tuberculosis* Δ*panCD* mutant, which requires pantothenic acid supplementation for *in vitro* growth. Importantly, this auxotrophic mutant is highly attenuated in mice, indicating that *M*. *tuberculosis* has virtually no access to pantothenic acid from the host during infection [24]. Thus, genetic complementation of the Δ*panCD* deletion from the episomal TetR expression vector should ensure plasmid stabilization during growth in a pantothenic acid-free environment even in the absence of antibiotic pressure. Toward this end, we first generated a c-*otsB2*-4×*tetO* knock-in mutant in a markerless *M*. *tuberculosis* Δ*panCD* mutant background via specialized transduction as described before (S2 Fig). Next, we constructed a plasmid in which the *panCD* operon from *M*. *tuberculosis* along with its native ribosome binding site was cloned downstream of the *tetR* gene under control of a single Hsp60 promoter, thereby transcriptionally coupling *panCD* and *tetR* expression. The resulting plasmid pMV261::*tetR*-G::*panCD* was subsequently transformed into the *M*. *tuberculosis* Δ*panCD*(u) c-*otsB2*-4×*tetO* knock-in mutant, yielding the *M*. *tuberculosis* Δ*panCD*(u) c-*otsB2*-tet-on strain. During culture in pantothenic acid-free media, this conditional mutant reproduced the relevant phenotypes of the *M*. *tuberculosis* c-*otsB2*-tet-on strain such as ATc-dependent growth (S8 Fig). Furthermore, the plasmid was stably maintained in this strain in the absence of antibiotic pressure even after extensive subculturing, suggesting that this conditional mutant was appropriate for testing in infection models.

**Fig 4:**
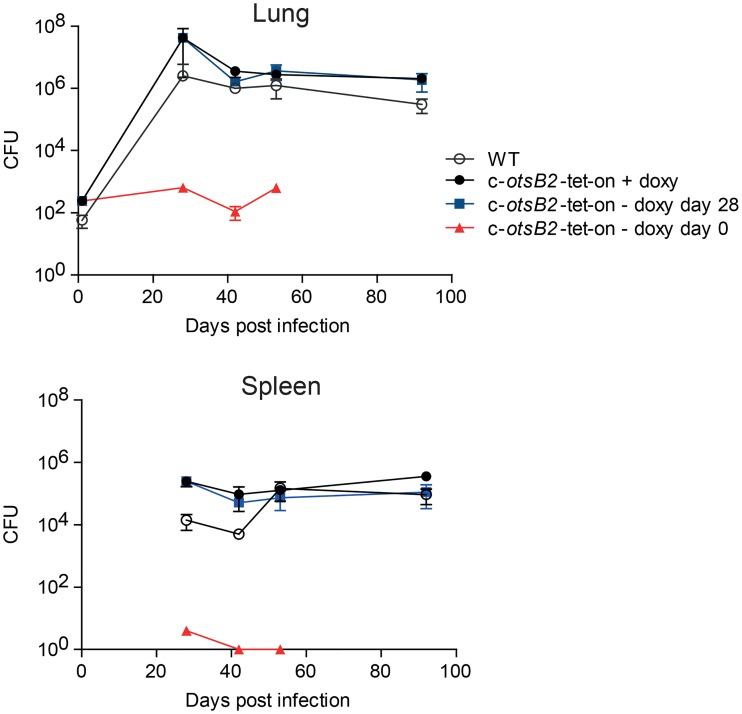
OtsB2 is required for *M*. *tuberculosis* to establish an acute infection in mice but dispensable for survival during the chronic phase. Mice were infected with *M*. *tuberculosis* strains via the aerosol route. Mice received doxycycline via the mouse chow to induce *otsB2* in the conditional *M*. *tuberculosis* c-*otsB2*-tet-on mutant. Doxycycline treatment was stopped either 24 h or 28 d post-infection to silence *otsB2* during the acute or chronic infection phases, respectively. Bacterial loads in lungs and spleens of C57BL/6 mice infected with *M*. *tuberculosis* H37Rv wild-type and the c-*otsB2*-tet-on mutant strain were determined by plating serial dilutions of organ homogenates on 7H10 agar containing 200 ng/ml ATc to determine viable bacterial cell counts. Aliquots were plated in parallel also on 7H10 agar containing no ATc to quantify the frequency of non-regulated suppressor mutants of the conditional c-*otsB2*-tet-on mutant, which was <1% at all time points and conditions. Data are means ± SD from four mice per group and time point (except c-*otsB2*-tet-on—doxy day 0, n = 3; at days 42 and 53). Data represent a single experiment. Silencing during the acute infection was repeated once to reproduce the bacteriostatic effect of OtsB2 inactivation (see S9 Fig).

To determine the role of OtsB2 for *M*. *tuberculosis* viability and virulence *in vivo*, mice were infected with the *M*. *tuberculosis* Δ*panCD*(u) c-*otsB2*-tet-on mutant via the aerosol route. Doxycycline provided in the food was used to induce *otsB2* expression. The *otsB2* gene was silenced by withdrawal of doxycycline either at day 0 or at day 28 post infection in order to assess the importance of OtsB2 for the acute or chronic infection phase, respectively (Fig 4). While induction of *otsB2* led to full virulence of the conditional mutant comparable to WT, silencing of *otsB2* following aerosol challenge resulted in an inability to establish an acute infection in mice and an inability of the bacterium to replicate *in vivo*. A bacteriostatic effect was observed in the lung with bacteria persisting at a very low organ burden after infection. Only a few bacteria disseminated to the spleen and were eventually eradicated from this organ. In contrast, when *otsB2* was silenced after day 28 when a chronic infection had already been established, no attenuation of the conditional mutant was detected in lungs and spleens. The efficiency of silencing during infection is difficult to be determined, and thus some residual *otsB2* expression in silenced cells cannot be ruled out. However, the presented data suggest that OtsB2 is much less important, if not fully dispensable, for the chronic infection phase in mice.

### Genome wide screen for synthetic lethal interactions

We have shown that the OtsA-OtsB2 pathway for trehalose biosynthesis appears to be much less active, if at all, during the chronic infection phase and that loss-of-function mutations in OtsA will mediate resistance against potential inhibitors of OtsB2. These observations reveal limitations to the drug target potential of OtsB2. We therefore performed a genome-wide screen to unravel synthetic lethal interactions with OtsA for the identification of additional targets, which would prevent resistance and result in synergistic killing when inhibited in combination with an OtsB2 inhibitor. For this, a saturated transposon mutant pool containing ~100,000 independent mutants was established in the *M*. *tuberculosis* Δ*otsA*(u) mutant in the presence of 500 μM trehalose. After subcultivation in the presence or absence of trehalose, the relative composition of the complex mutant libraries was analyzed using transposon insertion sequencing (TnSeq) [25].

By comparing the mutant libraries grown in the absence or presence of trehalose using a Hidden Markov Model method to identify loci with significantly different transposon insertion counts (see S1 Text), six genes were found to be differentially essential in the absence of trehalose (i.e. significantly fewer transposon insertions were detected in these genes in the Δ*otsA*(u) mutant background in absence of trehalose versus presence of trehalose) (Table 2). These included the genes *treX*, *treY* and *treZ*, as expected, since they mediate *de novo* synthesis of trehalose from α-glucans with their combined inactivation with *otsA* resulting in trehalose auxotrophy [12]. ADP-glucose pyrophosphorylase GlgC, which is involved in α-glucan production [26], was also found to be essential only in the absence of trehalose suggesting that inactivation of *glgC* in the Δ*otsA*(u) mutant results in glucan deficiency and depletes the TreX-TreY-TreZ pathway of its substrate. The nature of these genetic interactions implies that the two other identified genes, Rv0907 and Rv3160c of unknown function, might also play a direct or indirect role in α-glucan biosynthesis. Interestingly, one gene (Rv1845c) harbored significantly more transposon insertions in absence of trehalose. Thus, this gene, which is essential in WT [27], appears to become less essential in context of *otsA* deletion but only at low trehalose concentration. Rv1845c might be a sensor-transducer protein involved in sensing beta-lactams, but its precise function is unknown [28]. Thus, there is currently no obvious explanation for the differential gene essentiality and the nature of the interaction with OtsA.

**Table 2:** Genes differentially essential in the *M*. *tuberculosis* Δ*otsA* mutant in absence of trehalose supplementation. A saturated transposon mutant pool established in the *M*. *tuberculosis* Δ*otsA* mutant background was cultured either in the absence or presence of trehalose, subjected to transposon insertion sequencing (Tn-seq) and analyzed using a non-gene centric method. Genome areas harboring significantly less transposon insertions in absence of trehalose are shown. One genome area comprising most of gene Rv1845c harbored significantly more transposon insertions in absence of trehalose (i.e. appearing essential only in presence of trehalose) and is highlighted in grey.

Start nt	End nt	N of TA sites	Reads Δ*otsA* - trehalose	Reads Δ*otsA* + trehalose	p-value	Genes (% of ORF covered)	Segments type
1010144	1010572	10	0	140	0.00085	Rv0907 (25)	1
1010697	1011696	24	1	92	0.07207	Rv0907 (60)	1
1355864	1356654	28	69	591	0.00160	Rv1213-glgC (90)	2
1765353	1768480	55	1	1890	0.00000	Rv1561 (11) Rv1562c-treZ (100) Rv1563c treY (47)	1
1769196	1769305	4	0	51	0.03122	Rv1563c-treY (11)	1
1770109	1771117	25	5	200	0.00879	Rv1564c-treX (51)	1
1771157	1771634	13	19	522	0.00140	Rv1564c-treX (22)	2
2095257	2096106	14	115	0	0.00772	Rv1845c (93)	1
3529246	3529897	10	0	50	0.03894	Rv3160c (100)	1

In comparison to a published set of genes essential for *in vitro* growth of *M*. *tuberculosis* [27], 131 genes were found to be essential in the context of *otsA* deletion compared to WT irrespective of trehalose availability. Some of these may be due to use of different media (7H9 in this study versus minimal media in [27]) and might simply represent physiological differences rather than true differential genetic susceptibility based on lack of *otsA*. However, at least the observed effects on maltose-1-phosphate synthase *glgA* and components of a trehalose ABC transporter (*lpqY*, *sugA*, *sugC*) appear to be specific (S2 Table). We have very recently described the synthetic lethal interaction between *otsA* and *glgA* in *M*. *tuberculosis*, likely caused by toxic accumulation of the common precursor ADP-glucose [26]. Furthermore, trehalose recycling via the ABC transporter LpqY-SugABC becomes essential when the OtsA-OtsB2 pathway is blocked, likely by further depleting intracellular trehalose levels via secretion of trehalose during cell wall assembly.

## Discussion

Many potential candidates for target-based approaches in antibacterial drug discovery are selected only based on apparent genetic essentiality *in vitro*, while there is often a lack of knowledge of their relevance *in vivo* during different infection phases. The present study exemplifies that not only is *in vitro* gene essentiality a poor predictor of drug target suitability *in vivo* but also that the vulnerability of a target can change dramatically during the course of an infection. Thus, knowledge of the consequences of inactivation of potential targets during all relevant infection phases is imperative to make realistic predictions of target suitability and efficacy. Accordingly, we assessed OtsB2 as a drug target candidate in *M*. *tuberculosis* employing conditional gene silencing *in vitro* and in mice. Despite technical advances in recent years, the study of strictly essential genes in *M*. *tuberculosis* using conditional mutants is still a challenging endeavour, particularly in the context of animal infection models. So far, only 13 conditional *M*. *tuberculosis* mutants have been studied in mice (reviewed in [29]), but just seven of them were made for genes being strictly essential for *in vitro* growth. Six of these essential genes have been shown to be required during both the acute and the chronic infection phase in mice (*nadE* [30], *carD* [31], *pptT* [32], *pimA* [33], *bioA* [34], *glmU* [35]), while *ideR* is also essential for acute infection, but has not specifically been silenced during the chronic phase [36]. As revealed in this study, *otsB2* is the first example of an essential *M*. *tuberculosis* gene shown to be specifically required only during the acute infection phase in mice.

Differential OtsB2 essentiality during two infection stages indicates that trehalose metabolism of *M*. *tuberculosis* is substantially remodeled *in vivo*. The OtsA-OtsB2 pathway for trehalose biosynthesis dominates in culture and during the acute infection phase. In contrast, this pathway is surprisingly dispensable to maintain viability of *M*. *tuberculosis* during the chronic infection phase. This implies that flux through the OtsA-OtsB2 pathway is significantly reduced at later infection stages. This could be achieved by downregulating trehalose production altogether. However, there is evidence that the chronic infection phase in mice does not represent a static equilibrium of slow or nonreplicating bacilli. Rather, *M*. *tuberculosis* seems to continue replicating at least at basic level, while counteracting immune killing results in no net increase in organ burden of viable bacteria [37]. Since trehalose is essential for cell wall assembly during mycobacterial replication, trehalose biosynthesis probably continues at some rate also during chronic infection. Thus, it is more likely that the alternative TreX-TreY-TreZ pathway becomes activated during chronic infection to release trehalose from intracellular α-glucan storage molecules.

Silencing of *otsB2* in culture has a bactericidal effect whereas it is bacteriostatic *in vivo* during the acute infection phase. A likely explanation for this is a reduced flux through the OtsA-OtsB2 route *in vivo* compared to *in vitro* conditions, so that the level of toxic T6P that accumulates is somewhat less *in vivo*. Thus, while the OtsA-OtsB2 route clearly dominates during the acute infection phase, the alternative TreX-TreY-TreZ pathway might be already more active early during infection than under *in vitro* conditions. This is not unusual as we have recently described a similar *in vitro*-*in vivo* difference in flux through alternative sugar metabolic pathways for biosynthesis of maltose-1-phosphate in *M*. *tuberculosis* [26].

Our data conclusively show that essentiality of OtsB2 relies on direct or indirect toxic effects associated with OtsA-mediated T6P accumulation. While this apparent phosphosugar-related toxicity resembles a previous example in *M*. *tuberculosis* concerning M1P [19], we were surprised to observe a very different transcriptome profile in T6P-stressed cells, and little overlap with the stress response elicited by M1P. Two notable examples of a common signature in T6P- and M1P-stressed cells were the global upregulation of the *arg* operon required for *de novo* biosynthesis of L-arginine and induction of DNA damage-responsive genes including those of the SOS regulon [19]. Induction of arginine production has been described as a stress response upon hyperosmotic stress in yeast [38] and upon internalization of *Candida albicans* cells by macrophages [39]. Likewise, genes involved in arginine biosynthesis are also induced under hyperosmotic conditions in *M*. *tuberculosis* [40]. Therefore, it is conceivable that arginine may play a role as a general small-molecule stress protectant in *M*. *tuberculosis*, very similar to trehalose [41], to counteract various stresses that may be encountered in the host during infection. However, arginine could not confer any noticeable protective effects during stress associated with T6P accumulation, so this could represent a general, unspecific stress response that provides no advantage in this particular case. Further experiments involving *otsB2* silencing in arginine auxotrophic mutants are needed to address the role of arginine biosynthesis in phosphosugar stress response. T6P stress led to a strong upregulation of the putative efflux pump Rv1258c, which has been implicated in antibiotic resistance. However, this did not lead to a decreased susceptibility of stressed *M*. *tuberculosis* cells to the first-line anti-tuberculosis drugs isoniazid, rifampicin and ethambutol. While it cannot be ruled out that this efflux pump might be specific for other antibiotics, this suggests that OtsB2 inhibitors could be included in tuberculosis chemotherapy without impairing the efficacy of standard drugs. Whether upregulation of Rv1258c represents a specific mechanism to alleviate T6P stress or not is unknown. However, it is tempting to speculate that this could mediate efflux of T6P or downstream products to decrease the intracellular concentration. While we have not tested for potential secretion of T6P in silenced cells, the high levels of intracellular T6P accumulation implies that T6P cannot be exported at a substantial rate. Although not being overrepresented among the many genes induced in T6P stressed cells, upregulation of several DNA damage-inducible genes including those of the SOS regulon indicates that T6P accumulation might lead to DNA damage in a direct or indirect way, very similar to that described for M1P. While this probably explains the bactericidal effect of both T6P and M1P accumulation in *M*. *tuberculosis*, the molecular mechanisms of phosphosugar-mediated DNA damage are unclear in each case. Two intriguing features of the T6P-elicited transcriptome profile are the upregulation of *vapB* antitoxin genes, which might cause increased RNA half-life via enhanced neutralization of the RNase activity of VapC toxins whose specific RNA substrates have yet to be identified, and downregulation of highly-abundant non-coding RNAs of unknown functions. Whether these features contribute to the loss of viability upon OtsB2 inactivation or constitute responses that aim to mitigate and/or counteract the T6P stress needs to be addressed in the future.

The strict essentiality of OtsB2 to establish an acute infection in mice highlights that it might represent a new potential drug target candidate. However, conditional gene silencing *in vivo* has revealed several limitations of OtsB2 in terms of its suitability as a potential drug target. First, although the efficacy of gene silencing *in vivo* is unclear, it appears that inhibition of OtsB2 can maximally achieve bacteriostatic growth inhibition in the lung. Thus, monotherapy with OtsB2 inhibitors would not be sufficient to eradicate the pathogen, necessitating combination therapy with other drugs. Second, loss-of-function mutations in OtsA would abolish the efficacy of OtsB2 inhibitors by preventing T6P accumulation, thereby readily giving rise to resistance. And third, OtsB2 inhibitors would only be appropriate to treat acute infections because they become ineffective during the chronic infection phase. Nevertheless, a genome-wide screen for synthetic lethal interactions with OtsA has revealed several opportunities for the design of combination therapies that would suppress resistance and boost the efficacy of drugs targeting OtsB2. Since, in the absence of the OtsA-OtsB2 pathway, trehalose is synthesized via the TreX-TreY-TreZ pathway from α-glucans, targeting either the TreX-TreY-TreZ pathway or reactions required for α-glucan formation (e.g. GlgC, GlgA) in a combination therapy will prevent resistance that might arise through loss-of-function mutations in OtsA. Additionally, targeting both trehalose synthesis pathways simultaneously will likely enhance the growth inhibitory effect during the acute infection phase and might render *M*. *tuberculosis* cells susceptible towards OtsB2 inhibitors also during the chronic infection phase.

## Materials and Methods

### Strains and growth conditions

Cells of *M*. *tuberculosis* H37Rv were grown aerobically at 37°C in Middlebrook 7H9 medium supplemented with 10% (v/v) OADC enrichment (Becton Dickinson Microbiology Systems), 0.5% (v/v) glycerol and 0.05% (v/v) Tyloxapol. Hygromycin (50 mg/l), apramycin (20 mg/l) and kanamycin (20 mg/l) were added for selection for appropriate strains. All strains used for this study are listed in S3 Table.

### Generation of targeted gene deletion and conditional mutants

Mutants of *M*. *tuberculosis* were generated by allelic exchange using specialized transduction principally as described previously [42, 43]. Details on the generation of mutants and genetic complementation are described in S1 Text, using oligonucleotides listed in S4 Table for constructing specific allelic exchange substrates.

### Resazurin microplate assay (REMA) for growth quantification

Anhydrotetracycline (ATc)-dependent growth of conditional *M*. *tuberculosis otsB2* mutant strains was quantified using the resazurin microplate assay. Cells were first subcultured in 7H9 medium containing 100 ng/ml ATc for 7 days, harvested, and washed. A total volume of 100 μl medium per well in 96-well plates containing increasing concentrations of ATc (0–160 ng/ml) was then inoculated 1% with the washed cells and incubated for 6 days at 37°C. Subsequently, 10 μl resazurin solution (100 μg/ml, Sigma-Aldrich) was added and cells were incubated for further 16 h at 37°C. Then cells were fixed at room temperature for 30 min after addition of formalin (5%, v/v, final concentration) and fluorescence was measured using a microplate reader (excitation 560 nm, emission 590 nm).

### Thin-layer chromatography (TLC) and ^1^H-NMR analysis of carbohydrates

Carbohydrates were extracted from equal amounts of cells with hot water (95°C for 4 h) and analyzed by TLC on Silica gel 60 (EMD Chemicals) plates using the solvent system 1-propanol:ethyl acetate:water (6:1:3, v/v/v). Substances were visualized by immersing TLC plates in 10% (v/v) sulfuric acid in ethanol followed by charring at 180°C for 10 min. The extracts were also subjected to ^1^H-NMR spectroscopic analysis. Solution-state spectra were recorded on a Bruker Avance III 400 MHz spectrometer and analyzed using Bruker TopSpin 2.1 (Rheinstetten, Germany). Chemical shifts are reported with reference to residual water at δ_H_ 4.79 ppm and authentic trehalose and T6P were purchased from Sigma-Aldrich.

### Transcriptome profiling

Cells of the conditional *M*. *tuberculosis* c-*otsB2* mutant were grown from frozen stocks in 7H9 medium containing 1000 ng/ml ATc until they reached an OD_600 nm_ ~1. Cells were harvested, washed and then subcultured in 7H9 medium containing 100 ng/ml ATc for 7 days at 1% inoculation. Cells were subsequently harvested, washed, and used to inoculate 10–30 ml per replicate of 7H9 medium containing either 200 ng/ml or 30 ng/ml ATc, respectively, at 1% inoculation. Cells were harvested for RNA extraction after 7 days of incubation at 37°C, and cell pellets were fixed overnight in 5 ml RNA Protect reagent (Qiagen). Fixed cells were pelleted, resuspended in 1 ml RLT buffer (Qiagen) and lysed by bead beating to prepare lysates. Total RNA was extracted using the RNeasy Mini kit (Qiagen). Total RNA preparations were checked for RNA integrity by Agilent 2100 Bioanalyzer quality control. All samples in this study showed high quality RNA Integrity Numbers (RIN; mean 9.5). RNA was further analyzed by photometric Nanodrop measurement and quantified by fluorometric Qubit RNA assays (Life Technologies). After magnetic depletion of ribosomal RNA (Ribo-Zero rRNA Removal Kit, Gram-positive Bacteria; Epicentre), barcoded cDNA libraries were prepared according to the manufacturers protocol (Ion Total RNA-Seq Kit v2; Life Technologies). Emulsion PCR and subsequent IonProton sequencing were performed according to commercial kit protocols (Ion PI Template OT2 200 Kit v2, Ion PI Sequencing 200 Kit; Life Technologies). Demultiplexing was done using TorrentSuite software (vers. 4.0.2, Life Technologies). Raw sequencing reads were quality trimmed in CLC Genomics Workbench (vers. 6.5.2, CLCbio / Qiagen). After removal of short (< 50 nt) sequences, the remaining high quality reads were aligned against the *M*. *tuberculosis* H37Rv reference sequence NC_000962.3. RPKM [44] normalized read counts were log2 transformed for further analysis. Differential gene expression between two experimental conditions (three biological replicates each) was statistically determined by Student´s T-test (FDR corrected). The significance threshold was set to p(corr) = 0.01. RNAseq data have been deposited in the NCBI Gene Expression Omnibus (GEO Series accession number GSE70291).

### Quantitative real-time PCR

For qRT-PCR, DNA-free total RNA samples were prepared from independent biological replicates essentially as described above, with the omission of rRNA depletion, and were reverse transcribed with the SuperScript III First-Strand Synthesis System (Invitrogen). For the real-time reaction, each primer (250 nM) and 7.5 μl of template reaction (1:10 dilution) in 25 μl volume with GoTaq qPCR Master Mix (Promega) was used. Triplicate samples were run on a CFX96 Real-Time System (Bio-Rad). Threshold cycles were normalized to those for 16S rRNA. Primer sequences used for qRT-PCR are listed in S5 Table.

### Transposon insertion sequencing (Tn-seq)

Random mutagenesis of the *M*. *tuberculosis* Δ*otsA* mutant was done using a Himar-1 mariner transposon comprising a kanamycin-resistance cassette and the R6K origin of replication [45] with a hyperactive C9 Himar-1 transposase [46], where both elements were provided on the temperature-sensitive phage phAE180, employing specialized transduction essentially as reported previously [42]. Transposon mutants were selected on Middlebrook 7H10 agar in the presence of kanamycin (20 mg/l) and 500 μM trehalose. About 100,000 individual transposon mutants were pooled. The pool was subsequently subpassaged twice in liquid medium with or without 500 μM trehalose, and the relative genetic composition was analyzed using next-generation sequencing.

DNA samples extracted from the Tn-seq library grown with and without trehalose were prepared for sequencing (i.e. PCR amplification, adapter ligation) using a protocol described previously [25]. The samples were sequenced on an Illumina HiSeq 2500 instrument in paired-end mode, with a read length of 106 or 124 bp. Approximately one million read-pairs were collected for each sample. The raw reads were mapped to the reference genome, *M*. *tuberculosis* H37Rv (NC_000962.2), and read counts at each TA dinucleotide site were reduced to unique template counts using TPP in Transit [47]. The saturation of the samples (percent of TA sites in the genome represented) was 40.8% and 45.5%, and the mean template count at non-zero sites was 16 and 32.

A comparative analysis of the Δ*otsA* library (grown on 7H9) to a previous H37Rv *in-vitro* dataset [27] (grown in minimal-medium plus glycerol; 2 replicates, also mapped to H37Rv) was performed using the 'resampling' method in Transit [47], which detects genes with significant differences in insertion counts using a statistical permutation test. The template counts in the samples were normalized using the TTR method (trimmed total read-count), the difference in reads for each gene was compared to a null-distribution from 10,000 samples (permutations of the counts between the conditions) to derive a p-value, and the results were adjusted for multiple comparisons using the Benjamini-Hochberg procedure. Genes with significant differential essentiality were defined as those with an adjusted p-value less than 0.05.

A comparative analysis of the differentially essential regions in the Δ*otsA* library grown with versus without trehalose was performed as follows. Two Hidden Markov Models (HMMs) were used. One was designed to identify regions where there is a clear difference in that the TA sites have insertions in one condition but not the other. The second HMM was designed to identify regions of quantitative differential essentiality, in that the relative level of insertion counts is significantly lower (but not necessarily zero) in one condition than the other. The advantage of using HMMs to analyze Tn-seq data is that differentially essential regions can be identified in a non-gene-centric way, i.e. not restricted to ORF boundaries. First, a 3-state HMM was run on each dataset (independently) to label each TA site as either Essential (ES), Non-essential (NE), or Missing (MI). The objective of this HMM is to distinguish sites with zero insertions that are legitimate (i.e. due to biological selection) versus those that are likely missing from the library. The intended interpretation of the MI state is for isolated TA sites where no insertions were observed in the middle of otherwise non-essential regions. The HMM was implemented in Python using the Scipy library. The parameters of the HMM (priors, likelihood functions, transition probabilities) are given in S1 Text. Given a sequence of insertion counts at TA sites, the Viterbi algorithm is used to extract the most probable sequence of state labels for each site [48]. The sequence of TA sites in each dataset was then segmented into regions by comparing the state labels between the two conditions (with and without trehalose). "Type 1" segments were defined as regions labeled consecutively as essential (ES) in one dataset but not the other. A second HMM was implemented to detect regions of differential essentiality where there was a quantitative difference in the counts between the two conditions. The counts for the two datasets A[1..n] and B[1..n] were compared to produce an integer sequence as follows: +1 if A[i]>B[i], -1 if A[i]<B[i], else 0 (i.e. sgn(A[i]-B[i])). The objective of the second HMM was to identify regions where the counts were consistently biased toward one condition or the other by assigning unobservable state labels at each site, effectively smoothing over these discretized directional values. The directional HMM has 3 states: S1 for regions where counts in A are generally greater than B, S3 for regions where counts in A are generally less than B, and S2 for regions where A and B are either equal or alternate frequently. The parameters for this HMM are also given in S1 Text. The Viterbi algorithm was used to extract the state labeling for each site based on the most probable state sequence. "Type 2" segments were defined as maximal runs of consecutive TA sites labeled as S1 or as S3 by this HMM. To determine the statistical significance of the difference in counts between the two datasets in each of the type-1 and type-2 segments identified above, a permutation test was performed [47]. Insertion counts at TA sites in each segment were randomly permuted between the datasets 10,000 times to generate a null-distribution for the difference in the sum of the counts between the two datasets, and a p-value for the observed difference was calculated from this. The p-values were then adjusted for multiple comparisons by the Benjamini-Hochberg procedure, and a threshold of adjusted p values <0.05 was applied.

### Mouse infections

Aerosol infection of seven week old female C57BL/6 mice (Jackson Laboratory) was performed using an inhalation exposure system from Glas-Col and early log phase *M*. *tuberculosis* cultures prepared as single-cell suspensions in PBS to deliver 100–200 bacilli per mouse. Four mice were sacrificed per strain per time point. Serial dilutions of lung and spleen homogenates were plated on appropriate 7H10 agar plates, that had been supplemented with 500 ng/ml ATc, 20 μg/ml kanamycin and 50 μg/ml hygromycin for the conditional c-*otsB2*-tet-on mutant. Aliquots of the conditional c-*otsB2*-tet-on mutant were plated in parallel on agar containing 20 μg/ml kanamycin and 50 μg/ml hygromycin but no ATc to determine the frequency of non-regulated suppressor mutants.

### Ethics statement

Mouse studies were performed in accordance to National Institutes of Health guidelines using recommendations in the Guide for the Care and Use of Laboratory Animals. Procedures involving mice were reviewed and approved by the Institutional Animal Care and Use Committee of Weill Cornell Medical College (protocol number 0601-441A).

## Supporting Information

S1 Fig Generation of *M*. *tuberculosis otsB2* gene deletion mutants. (A) Organization of the *otsB2* locus in *M*. *tuberculosis* wild-type as well as in a marked *otsB2* gene deletion mutant. The sizes of relevant fragments as well as the location of the probe used for Southern analyses are indicated. WT, wild-type; (u), unmarked locus; γδr*es*, *res*-sites of the γδ-resolvase; *hyg*, hygromycin resistance gene; *sacB*, levansucrase gene from *Bacillus subtilis*. (B) Southern analyses of *Pvu*I-digested genomic DNA using a probe hybridizing to the position indicated in A, showing *otsB2* gene deletion in an *otsB2* merodiploid strain (left) and in an unmarked *otsA* mutant.
(TIF)

S2 Fig Generation of *M*. *tuberculosis* c-*otsB2*-4×*tetO* gene knock-in mutants. (A) Organization of the *otsB2* locus in *M*. *tuberculosis* wild-type as well as in a c-*otsB2*-4×*tetO* gene knock-in mutant. The sizes of relevant fragments as well as the location of the probe used for Southern analyses are indicated. WT, wild-type; (u), unmarked locus; *hyg*, hygromycin resistance gene; Pmyc1-4×*tetO*, promoter cassette containing 4 *tetO* sites. (B) Southern analyses of *Sma*I-digested genomic DNA using a probe hybridizing to the position indicated in A, showing promoter cassette insertion in wild-type (upper left panel), in a Δ*panCD* gene deletion mutant (upper right panel), and in an unmarked *otsA* gene deletion mutant (lower left panel).
(TIF)

S3 Fig Generation of a marked and unmarked *M*. *tuberculosis otsA* gene deletion mutants. (A) Organization of the *otsA* locus in *M*. *tuberculosis* wild-type as well as in a marked and unmarked *otsA* gene deletion mutant. The sizes of relevant fragments as well as the location of the probe used for Southern analyses are indicated. WT, wild-type; (u), unmarked locus; γδr*es*, *res*-sites of the γδ-resolvase; *hyg*, hygromycin resistance gene; *sacB*, levansucrase gene from *Bacillus subtilis*. (B) Southern analyses of *Eco*RV-digested genomic DNA using a probe hybridizing to the position indicated in A, showing *otsA* gene deletion and marker cassette removal.
(TIF)

S4 Fig Exogenous T6P has no toxic effect on *M*. *tuberculosis* cells. (A) WT cells were grown in liquid medium containing increasing concentrations of T6P, revealing no growth inhibitory effect. (B) Cells of the conditional *M*. *tuberculosis* c-*otsB2*-tet-on mutant were cultivated in liquid medium containing increasing concentrations of ATc either in presence or absence of 1 mM T6P. ATc-dependent growth was not substantially altered by the presence of T6P, revealing no toxic effect of exogenous T6P. Growth in A and B was determined employing the resazurin microplate assay. Values are means of triplicates ± SEM.
(TIF)

S5 Fig Drug susceptibility of induced and partially silenced cells of the conditional *M*. *tuberculosis* c-*otsB2*-tet-on mutant. Cells of the conditional *M*. *tuberculosis* c-*otsB2*-tet-on mutant were either induced in presence of 200 ng/ml or partially silenced in presence of 30 ng/ml ATc and incubated with the indicated concentrations of either rifampicin, isoniazid, or ethambutol for 5 days. Growth was determined employing the resazurin microplate assay using non-inoculated medium (0% growth) and solvent treated cells (DMSO; 100% growth) as controls. WT, wild-type.
(TIF)

S6 Fig Arginine supplementation does not rescue growth of the conditional *M*. *tuberculosis* c-*otsB2*-tet-on mutant under silencing conditions. Cells of the conditional *M*. *tuberculosis* c-*otsB2*-tet-on mutant were cultivated in liquid medium containing increasing concentrations of ATc either in presence or absence of 1 mM arginine. ATc-dependent growth was not substantially altered by the presence of arginine, revealing no stress-protective effect of exogenous arginine during T6P accumulation. Growth was determined employing the resazurin microplate assay. Values are means of triplicates ± SEM.
(TIF)

S7 Fig Differential gene expression of the DNA-damage-responsive genes in induced and partially silenced cells of the conditional *M*. *tuberculosis* c-*otsB2*-tet-on mutant. Cells were cultivated either in presence of 200 ng/ml (100% growth relative to WT) or 30 ng/ml ATc (ca. 30% residual growth relative to WT) for 7 days. rRNA-depleted samples were analyzed by RNAseq. Genes with corrected p-values <0.01 are shown in red. The data indicate global upregulation of DNA damage responsive genes including those of the SOS regulon in partially silenced, trehalose-6-phosphate stressed cells. DNA damage-responsive genes in *M*. *tuberculosis* have been defined as those that respond to DNA damage as a result of treatment with DNA damaging agents such as fluorochinolones, UV irradiation, H_2_O_2_, and mitomycin C [21].
(TIF)

S8 Fig Anhydrotetracycline- (ATc-) dependent growth of the conditional *M*. *tuberculosis* Δ*panCD* c-otsB2-tet-on mutant. Growth of three independent clones was measured using the resazurin microplate assay.
(TIF)

S9 Fig Independent silencing experiment to reproduce the bacteriostatic effect of *otsB2* silencing during the acute infection phase in mice. Mice were infected with the conditional *M*. *tuberculosis* c-*otsB2*-tet-on mutant via the aerosol route. Mice received doxycycline via the mouse chow to induce *otsB2* in the conditional *M*. *tuberculosis* c-*otsB2*-tet-on mutant. Doxycycline treatment was stopped in one group 24 h post-infection to silence *otsB2* during the acute infection phase. Bacterial loads in lungs of infected C57BL/6 mice were determined by plating serial dilutions of organ homogenates on 7H10 agar containing 200 ng/ml ATc to determine viable bacterial cell counts. Aliquots were plated in parallel also on 7H10 agar containing no ATc to quantify the frequency of non-regulated suppressor mutants of the conditional c-*otsB2*-tet-on mutant, which was <1% at all time points and conditions. Data are means ± SD from four mice per group and time point. See also Fig 4.
(TIF)

S1 Text Supplementary Material and Methods. (PDF)

S1 Table Most abundant transcripts in induced and partially silenced cells of the conditional *M*. *tuberculosis* c-*otsB2*-tet-on mutant. Cells were cultivated in presence of 200 ng/ml (100% growth relative to WT) or 30 ng/ml ATc (ca. 30% residual growth relative to WT) for 7 days. rRNA-depleted samples were analyzed by RNAseq. RPKM, reads per kilobase of transcript per million mapped reads.
(PDF)

S2 Table Differentially essential genes in the *M*. *tuberculosis* Δ*otsA* mutant compared to wild-type. In order to identify those genes in the *M*. *tuberculosis* Δ*otsA* mutant background whose inactivation cannot be rescued by supplementation with trehalose, i.e. those genes that are essential in context of *otsA* deletion both in absence and presence of trehalose but non-essential in WT, a saturated transposon mutant pool was generated in the *M*. *tuberculosis* Δ*otsA* mutant background, cultured in the absence of trehalose and subjected to transposon insertion sequencing (Tn-seq). Genes harboring significantly less transposon insertions compared to a transposon mutant library established in *M*. *tuberculosis* H37Rv wild-type [27] are shown (p<0.05). Few genes harboring significantly more transposon insertions compared to a transposon mutant library established in *M*. *tuberculosis* H37Rv wild-type [27] (i.e. genes appearing less essential in context of *otsA* gene deletion) are highlighted in grey.
(PDF)

S3 Table Strains of *M*. *tuberculosis* H37Rv used in this study. Mutants were generated by allelic exchange employing specialized transduction as described in S1 Text. Abbreviations: WT, wild-type; Kan^r^, kanamycin resistant; Hyg^r^, hygromycin resistant; Apra, apramycin resistant; _u_, unmarked mutant.
(PDF)

S4 Table Oligonucleotides used for generation of allelic exchange substrates. Resulting phages listed here were used for generation of gene deletion or knock-in mutants of *M*. *tuberculosis* H37Rv listed in S3 Table by specialized transduction as described in S1 Text.
(PDF)

S5 Table Oligonucleotides used for qRT-PCR of *M*. *tuberculosis* transcripts. (PDF)

S1 Dataset RNAseq analysis of the stress response elicited by trehalose-6-phosphate accumulation. (XLSX)
