# Supplementary figures and images for: Trehalose-6-Phosphate-Mediated Toxicity Determines Essentiality of OtsB2 in Mycobacterium tuberculosis In Vitro and in Mice
Source: PLoS Pathog. 2016 Dec 9;12(12):e1006043. doi: 10.1371/journal.ppat.1006043 (PMC5148154; doi:10.1371/journal.ppat.1006043)

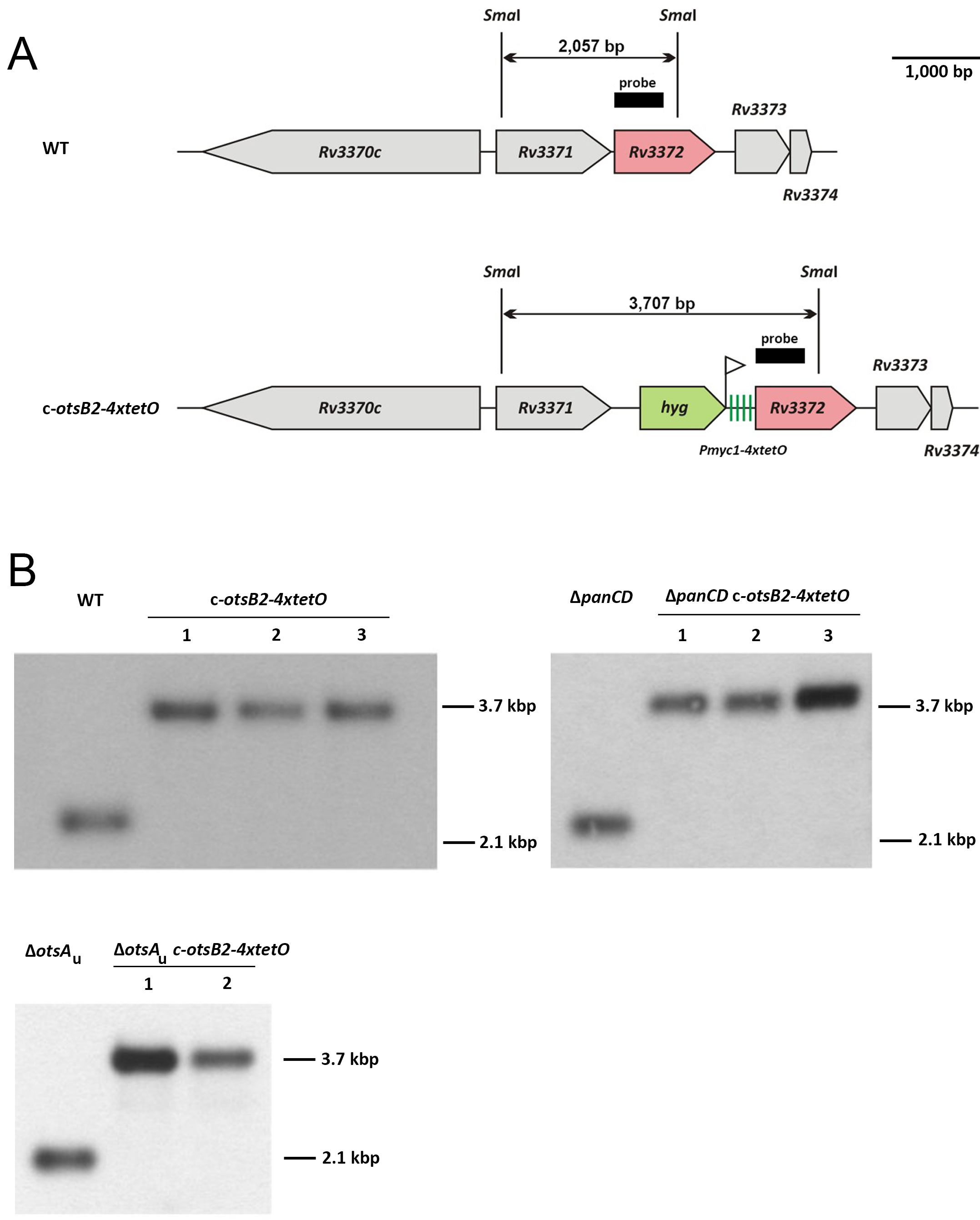

Supplement: S1 Fig — (A) Organization of the otsB2 locus in M. tuberculosis wild-type as well as in a marked otsB2 gene deletion mutant. The sizes of relevant fragments as well as the location of the probe used for Southern analyses are indicated. WT, wild-type; (u), unmarked locus; γδres, res-sites of the γδ-resolvase; hyg, hygromycin resistance gene; sacB, levansucrase gene from Bacillus subtilis. (B) Southern analyses of PvuI-digested genomic DNA using a probe hybridizing to the position indicated in A, showing otsB2 gene deletion in an otsB2 merodiploid strain (left) and in an unmarked otsA mutant. (TIF) [file ppat.1006043.s001.tif]

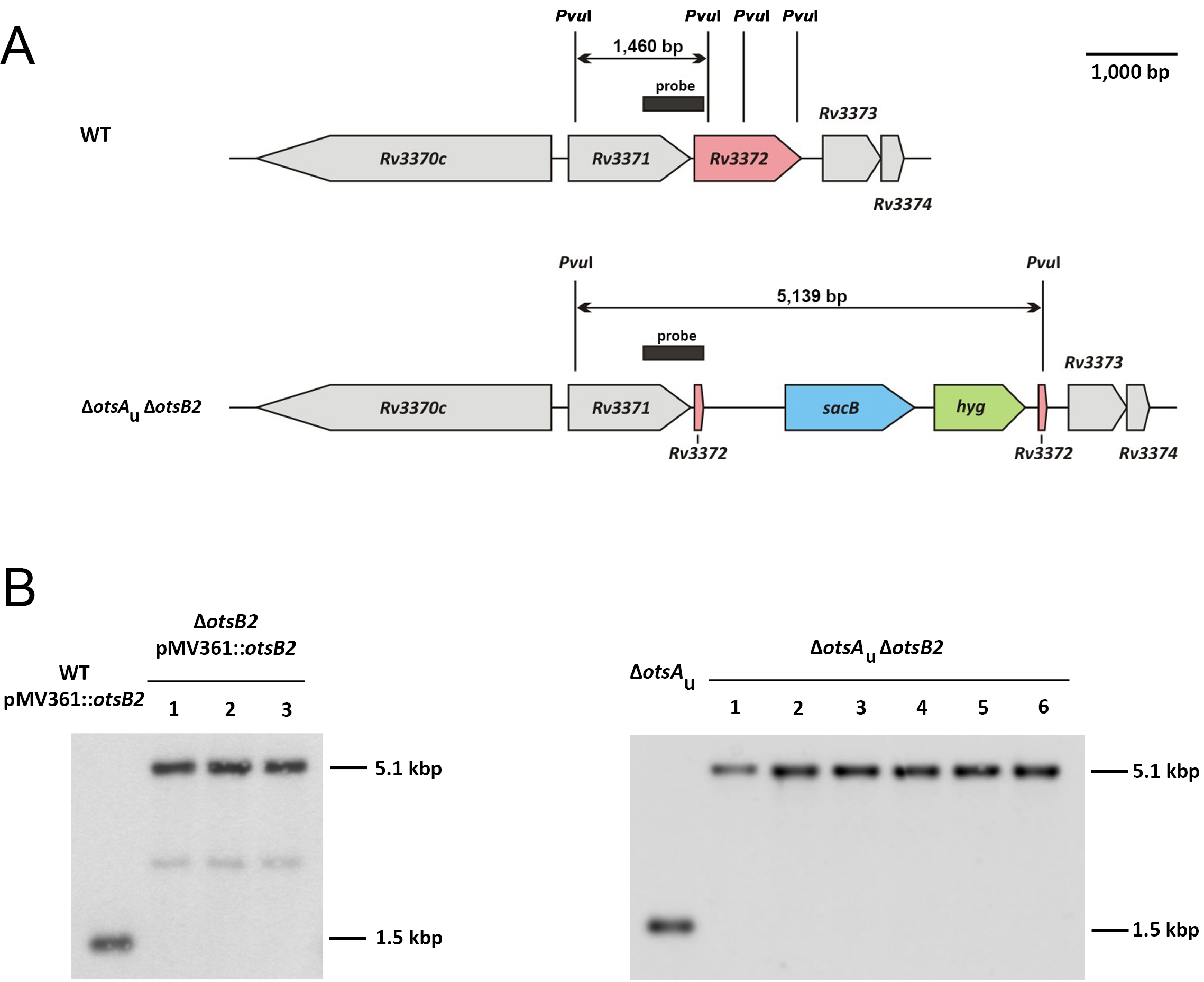

Supplement: S2 Fig — (A) Organization of the otsB2 locus in M. tuberculosis wild-type as well as in a c-otsB2-4×tetO gene knock-in mutant. The sizes of relevant fragments as well as the location of the probe used for Southern analyses are indicated. WT, wild-type; (u), unmarked locus; hyg, hygromycin resistance gene; Pmyc1-4×tetO, promoter cassette containing 4 tetO sites. (B) Southern analyses of SmaI-digested genomic DNA using a probe hybridizing to the position indicated in A, showing promoter cassette insertion in wild-type (upper left panel), in a ΔpanCD gene deletion mutant (upper right panel), and in an unmarked otsA gene deletion mutant (lower left panel). (TIF) [file ppat.1006043.s002.tif]

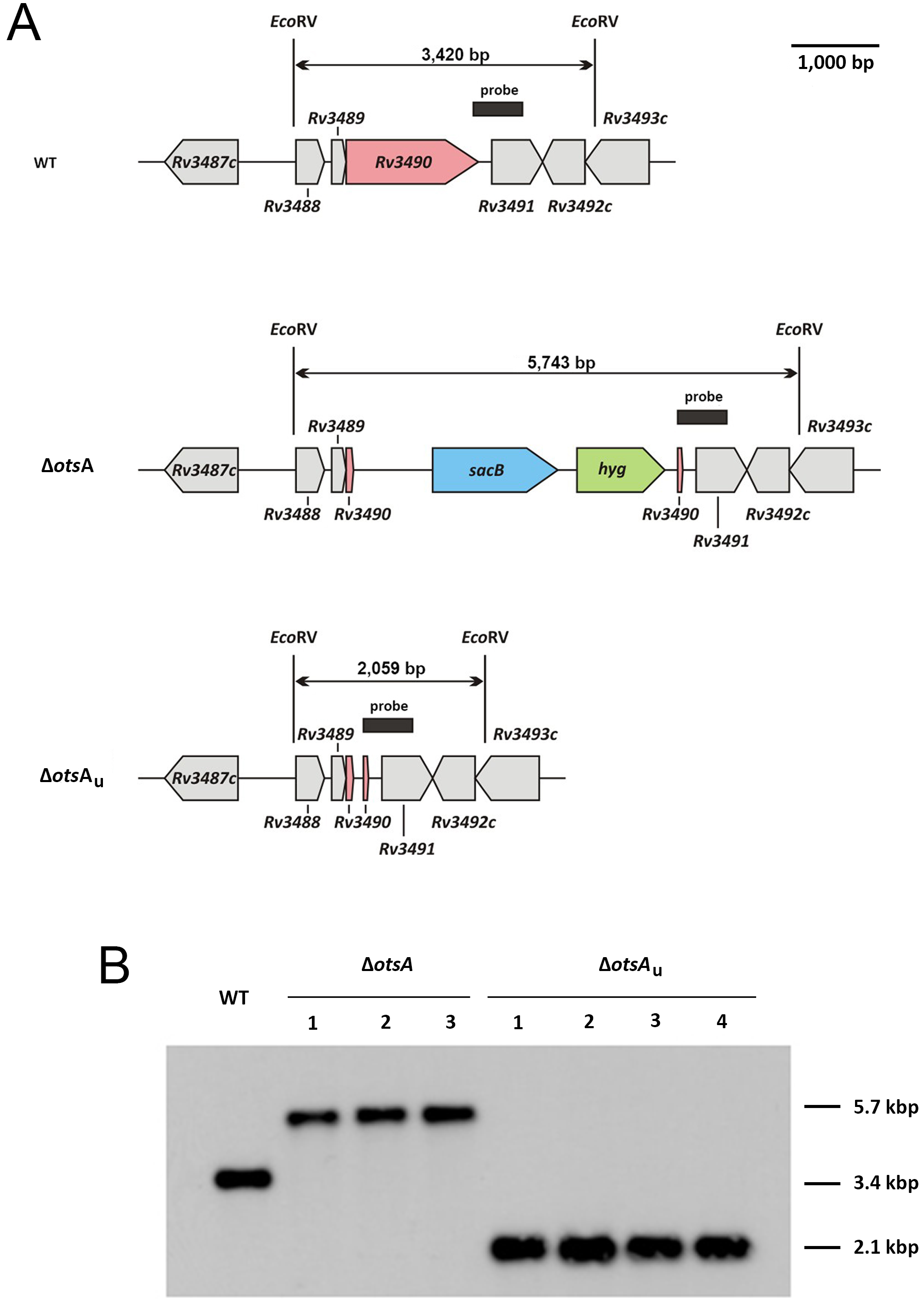

Supplement: S3 Fig — (A) Organization of the otsA locus in M. tuberculosis wild-type as well as in a marked and unmarked otsA gene deletion mutant. The sizes of relevant fragments as well as the location of the probe used for Southern analyses are indicated. WT, wild-type; (u), unmarked locus; γδres, res-sites of the γδ-resolvase; hyg, hygromycin resistance gene; sacB, levansucrase gene from Bacillus subtilis. (B) Southern analyses of EcoRV-digested genomic DNA using a probe hybridizing to the position indicated in A, showing otsA gene deletion and marker cassette removal. (TIF) [file ppat.1006043.s003.tif]

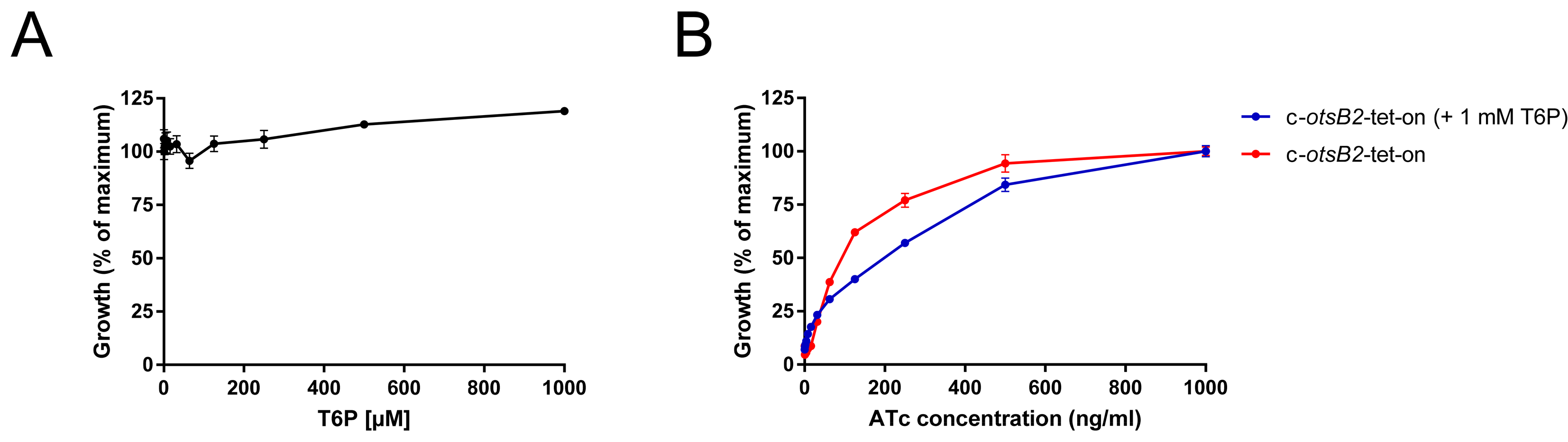

Supplement: S4 Fig — (A) WT cells were grown in liquid medium containing increasing concentrations of T6P, revealing no growth inhibitory effect. (B) Cells of the conditional M. tuberculosis c-otsB2-tet-on mutant were cultivated in liquid medium containing increasing concentrations of ATc either in presence or absence of 1 mM T6P. ATc-dependent growth was not substantially altered by the presence of T6P, revealing no toxic effect of exogenous T6P. Growth in A and B was determined employing the resazurin microplate assay. Values are means of triplicates ± SEM. (TIF) [file ppat.1006043.s004.tif]

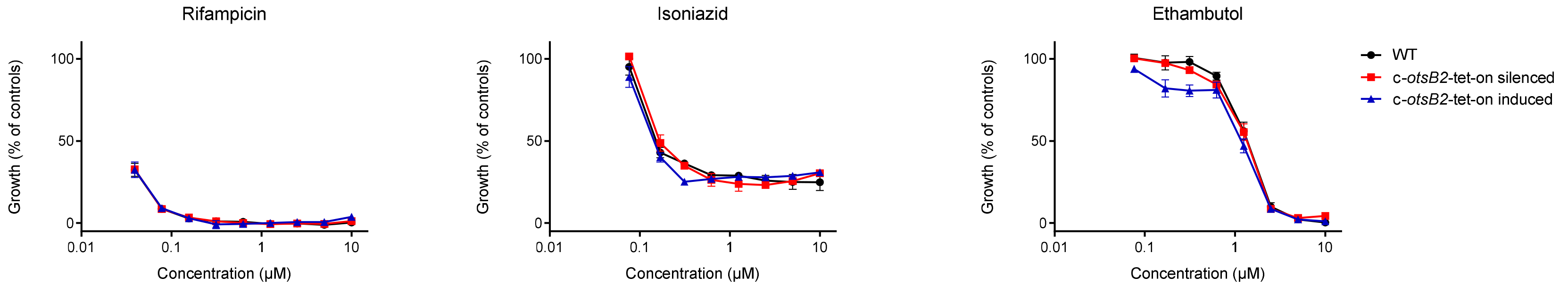

Supplement: S5 Fig — Cells of the conditional M. tuberculosis c-otsB2-tet-on mutant were either induced in presence of 200 ng/ml or partially silenced in presence of 30 ng/ml ATc and incubated with the indicated concentrations of either rifampicin, isoniazid, or ethambutol for 5 days. Growth was determined employing the resazurin microplate assay using non-inoculated medium (0% growth) and solvent treated cells (DMSO; 100% growth) as controls. WT, wild-type. (TIF) [file ppat.1006043.s005.tif]

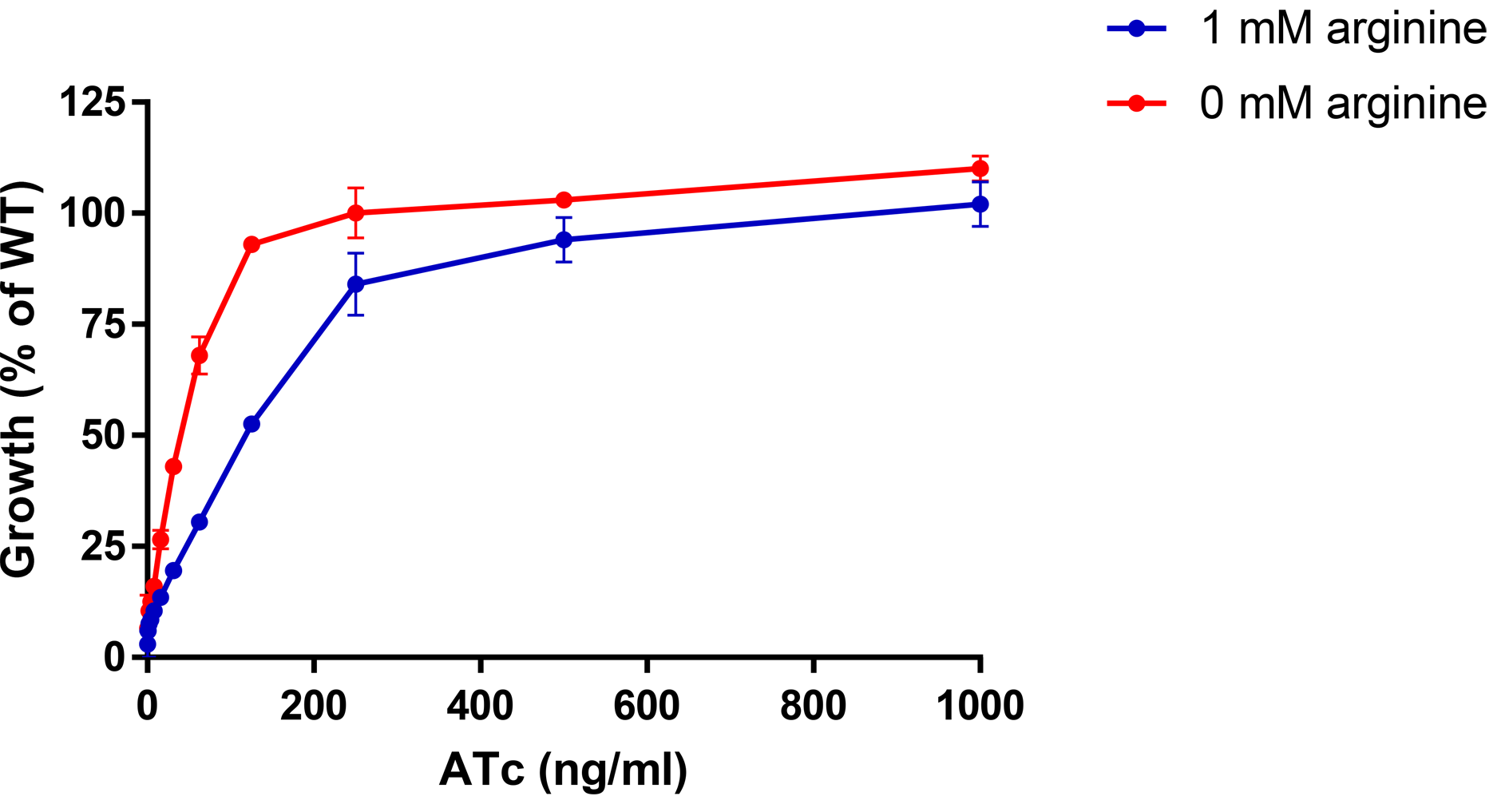

Supplement: S6 Fig — Cells of the conditional M. tuberculosis c-otsB2-tet-on mutant were cultivated in liquid medium containing increasing concentrations of ATc either in presence or absence of 1 mM arginine. ATc-dependent growth was not substantially altered by the presence of arginine, revealing no stress-protective effect of exogenous arginine during T6P accumulation. Growth was determined employing the resazurin microplate assay. Values are means of triplicates ± SEM. (TIF) [file ppat.1006043.s006.tif]

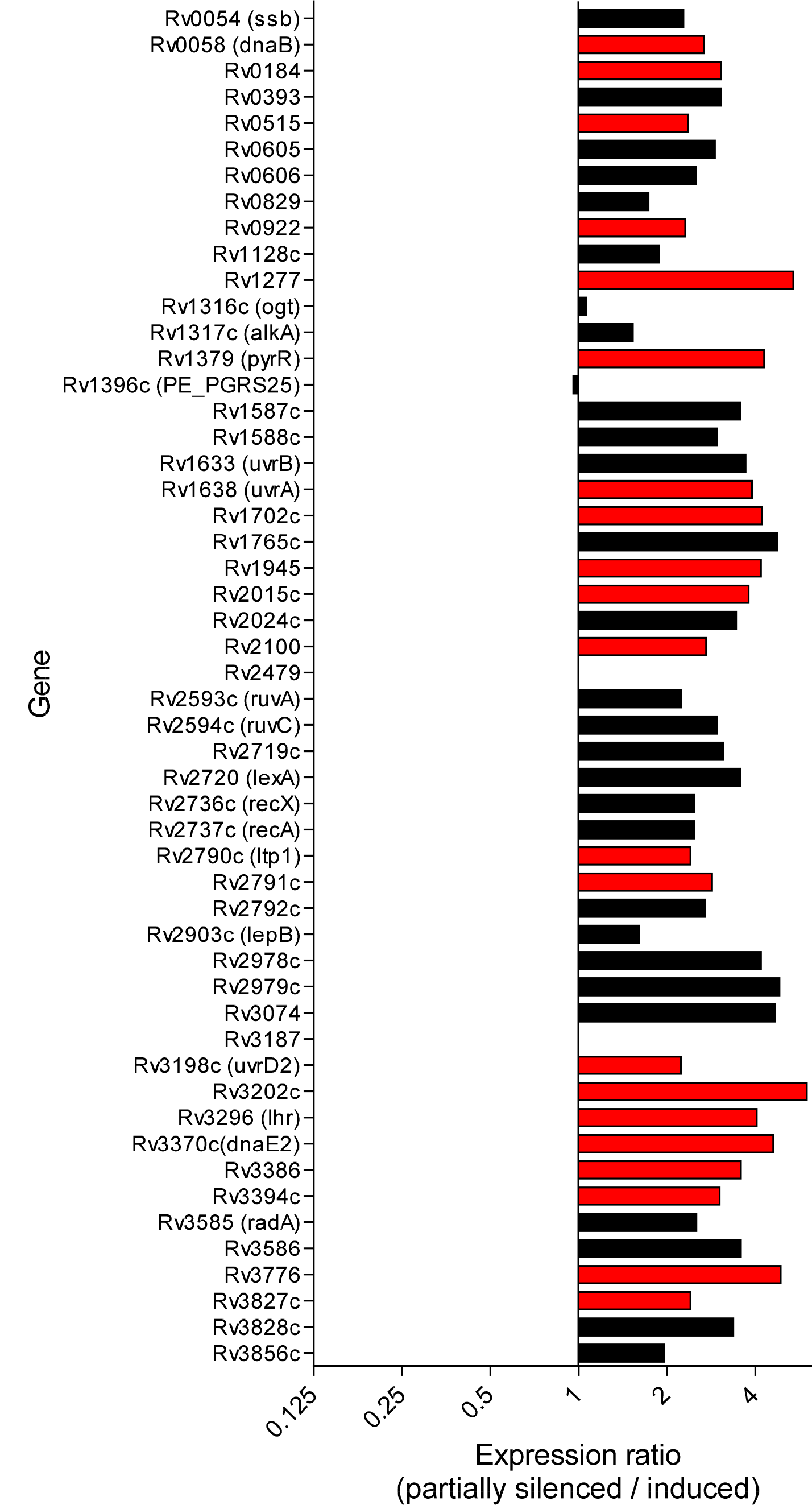

Supplement: S7 Fig — Cells were cultivated either in presence of 200 ng/ml (100% growth relative to WT) or 30 ng/ml ATc (ca. 30% residual growth relative to WT) for 7 days. rRNA-depleted samples were analyzed by RNAseq. Genes with corrected p-values <0.01 are shown in red. The data indicate global upregulation of DNA damage responsive genes including those of the SOS regulon in partially silenced, trehalose-6-phosphate stressed cells. DNA damage-responsive genes in M. tuberculosis have been defined as those that respond to DNA damage as a result of treatment with DNA damaging agents such as fluorochinolones, UV irradiation, H2O2, and mitomycin C [21]. (TIF) [file ppat.1006043.s007.tif]

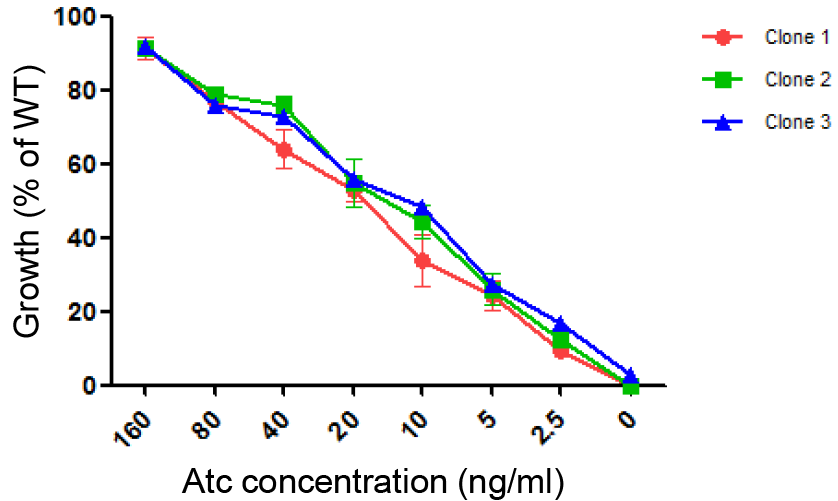

Supplement: S8 Fig — Growth of three independent clones was measured using the resazurin microplate assay. (TIF) [file ppat.1006043.s008.tif]

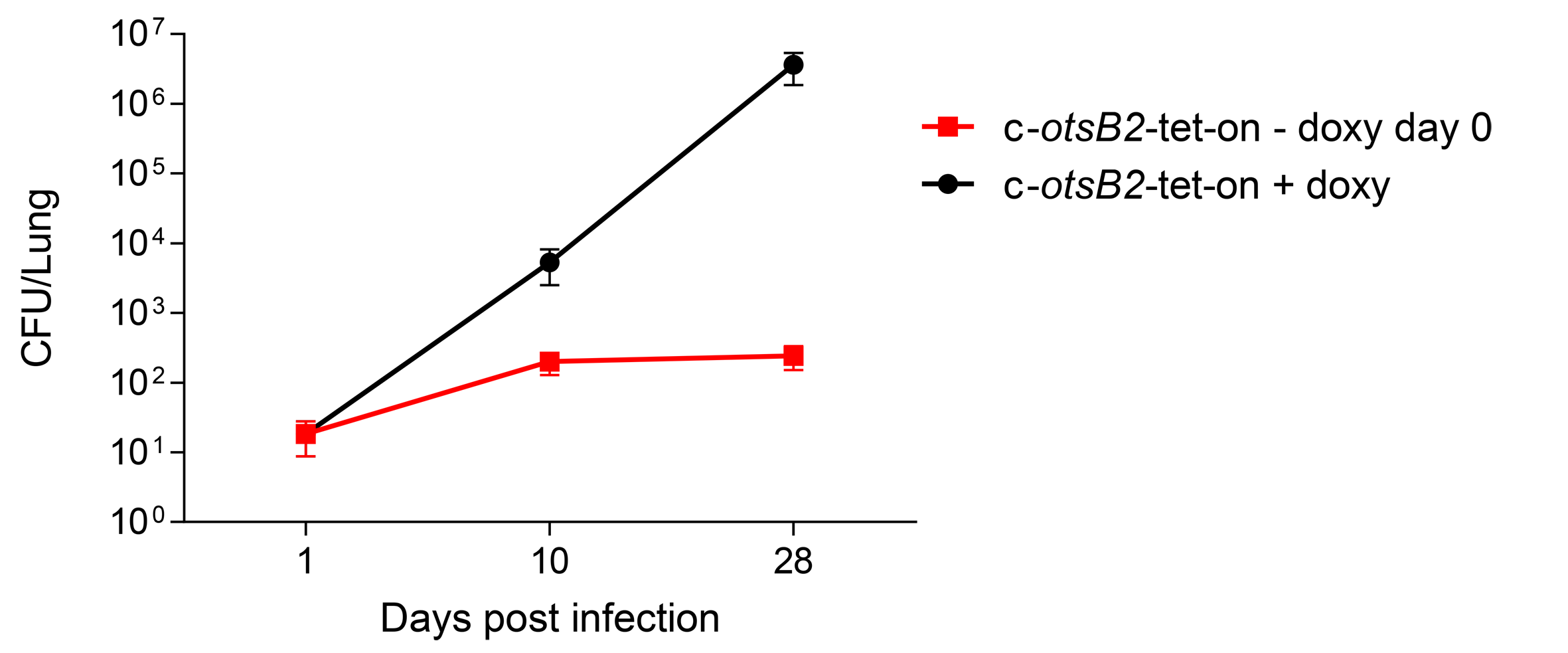

Supplement: S9 Fig — Mice were infected with the conditional M. tuberculosis c-otsB2-tet-on mutant via the aerosol route. Mice received doxycycline via the mouse chow to induce otsB2 in the conditional M. tuberculosis c-otsB2-tet-on mutant. Doxycycline treatment was stopped in one group 24 h post-infection to silence otsB2 during the acute infection phase. Bacterial loads in lungs of infected C57BL/6 mice were determined by plating serial dilutions of organ homogenates on 7H10 agar containing 200 ng/ml ATc to determine viable bacterial cell counts. Aliquots were plated in parallel also on 7H10 agar containing no ATc to quantify the frequency of non-regulated suppressor mutants of the conditional c-otsB2-tet-on mutant, which was <1% at all time points and conditions. Data are means ± SD from four mice per group and time point. See also Fig 4. (TIF) [file ppat.1006043.s009.tif]
