# Supplementary material for: Trehalose-6-Phosphate-Mediated Toxicity Determines Essentiality of OtsB2 in Mycobacterium tuberculosis In Vitro and in Mice
Source: PLoS Pathog. 2016 Dec 9;12(12):e1006043. doi: 10.1371/journal.ppat.1006043 (PMC5148154; doi:10.1371/journal.ppat.1006043)
Supplement: S1 Text — (PDF) [file ppat.1006043.s010.pdf]

S1 Text

Generation of site-specific gene deletion mutants. Site-specific *otsA* and *otsB2* gene deletion mutants of *M. tuberculosis* H37Rv were generated by specialized transduction employing temperature-sensitive mycobacteriophages essentially as described previously [1, 2]. Briefly, for generation of allelic exchange constructs for gene replacement with a γδ*res*-*sacB*-*hyg*-γδ*res* cassette comprising a *sacB* as well as a hygromycin resistance gene flanked by *res*-sites of the γδ-resolvase, upstream- and downstream-flanking DNA regions were amplified by PCR employing the oligonucleotides listed in **S5 Table**. Subsequently, the upstream and downstream flanks were digested with the indicated restriction enzymes, and ligated with *Van*91I-digested pYUB1471 vector arms [3]. The resulting knock-out plasmids were then linearized with *Pac*I and cloned and packaged into the temperature-sensitive phage phAE159 [3], yielding knock-out phages which were propagated in *M. smegmatis* at 30 °C. Allelic exchange in *M. tuberculosis* using the knock-out phages was achieved by specialized transduction using hygromycin (50 mg/l) for selection, resulting in gene deletion and replacement by the γδ*res*-*sacB*-*hyg*-γδ*res* cassette. For the generation of unmarked *otsA* mutants, the γδ*res*-*sacB*-*hyg*-γδ*res* cassette was removed employing specialized transduction using the phage phAE280 expressing the γδ-resolvase [3] using sucrose (3%, w/v) for counterselection. All obtained mutants were verified by Southern analysis of digested genomic DNA using appropriate restriction enzymes and probes (**S1 Fig** + **S3 Fig**).

Generation of the conditional *M. tuberculosis* c-*otsB2*-tet-on mutant. For establishing regulated expression of the *otsB2* gene, a synthetic gene cassette (*hyg*-P*myc1*-4X*tetO*) comprising a hygromycin resistance gene and the P*myc1* promoter from *M. smegmatis* engineered to contain four *tetO* operator sites, which are the DNA binding sites for the cognate repressor protein TetR, was inserted immediately upstream of the *otsB2* start codon in *M. tuberculosis*. Targeted gene knock-in was achieved by specialized transduction employing temperature-sensitive mycobacteriophages essentially as described above for gene deletion

mutants. Briefly, for generation of allelic exchange constructs for site-specific insertion in *M. tuberculosis* of the *hyg*-P*myc1*-4X*tetO* cassette, upstream- and downstream DNA regions flanking the *otsB2* start codon were amplified by PCR employing the oligonucleotides listed in **S5 Table**. Subsequently, the upstream and downstream flanks were digested with the indicated restriction enzymes, and ligated with *Van*91I-digested pcRv1327c-4XtetO vector arms [4]. The resulting knock-in plasmid was then linearized with *Pac*I and cloned and packaged into the temperature-sensitive phage phAE159 [3], yielding a knock-in phage which was propagated in *M. smegmatis* at 30°C. Allelic exchange in *M. tuberculosis* using the knock-in phage at the nonpermissive temperature of 37°C was achieved by specialized transduction using hygromycin (50 mg/l) for selection, resulting in site-specific insertion of the *hyg*-P*myc1*-4X*tetO* cassette. The obtained *M. tuberculosis* c-*otsB2*-4×*tetO* knock-in mutant was verified by Southern analysis of digested genomic DNA using an appropriate restriction enzyme and probe (**S2 Fig**).

For achieving controlled gene expression of the *otsB2* gene, the *E. coli* Tn10 *tetR* gene encoding a repressor protein exhibiting high-binding affinity to *tetO* sites in absence of the inducer tetracycline was heterologously expressed in the knock-in mutant. The *tetR* gene was amplified by PCR employing the oligonucleotide primer pair 5´-TTTTTT<u>GAATTC</u>ATGATGTCTAGATTAGATAAAAG-3´ and 5´-TTTTTT<u>AAGCTT</u>AAGACCCACTTTCACATTTAAG-3´ using an irrelevant *tetR*-harboring plasmid as a template and cloned using the restriction enzymes *EcoR*I and *Hin*dIII (underlined) into the episomal *E. coli*-mycobacterium shuttle plasmid pMV261-RBS-G, which is a derivative of plasmid pMV261 [5] harboring a mutated ribosome binding site [4]. The resulting plasmid pMV261::*tetR*-G providing constitutive gene expression from the HSP60 promoter in mycobacteria was transformed by electroporation into the *M. tuberculosis* c-*otsB2*-4x*tetO* knock-in mutant using solid medium containing 50 mg/l hygromycin and 20 mg/l kanamycin for selection. This yielded the conditional mutant *M. tuberculosis* c-*otsB2*-4x*tetO* pMV261::*tetR*-G (referred to as *M. tuberculosis* c-*otsB2*-tet-on mutant) allowing silencing of the *otsB2* gene in absence of the inducer anhydrotetracycline (ATc).

For regulated gene expression of *otsB2* in the Δ*panCD* mutant background, the *panC-panD* operon comprising the native ribosome binding sites was amplified by PCR employing the oligonucleotide primer pair 5´-TTTTTAAGCTTGAGGTTTTGACGGCATGACGATTC-3´ and 5´-TTTTTAAGCTTCTATCCCACACCGAGCCGGGGGTC-3´ using wild-type *M. tuberculosis* H37Rv genomic DNA as a template and cloned using the restriction enzyme *Hin*dIII (underlined) into plasmid pMV261::*tetR*-G downstream of the *tetR* gene in collinear orientation, thereby establishing transcriptional coupling of the *tetR* and *panCD* genes all being expressed from the HSP60 promoter. The resulting plasmid pMV261::*tetR*-G::*panCD* was transformed by electroporation into the *M. tuberculosis* Δ*panCD* c-*otsB2*-4x*tetO* knock-in mutant using solid medium containing 50 mg/l hygromycin, 20 mg/l kanamycin and no pantothenic acid supplementation for selection. This yielded the conditional mutant *M. tuberculosis* Δ*panCD* c-*otsB2*-4x*tetO* pMV261::*tetR*-G::*panCD* (referred to as *M. tuberculosis* Δ*panCD* c-*otsB2*-tet-on mutant).

Genetic complementation. For complementation of the conditional *M. tuberculosis* c-*otsB2*-tet-on mutant and for generating an *otsB2* merodiploid strain, the *otsB2* gene was PCR amplified using the oligonucleotide pair 5´-TTTTTTTAATTAAGTGCGCAAGTTGGGCCCGGTC-3' and 5'-TTTTTAAGCTTTCACGTTGCCCGCAGGGGAGC-3' and cloned using the restriction enzymes *Pac*I and *Hin*dIII (underlined) into the single-copy integrative plasmids pMV361(Apra)-PacI or pMV361(Kan)-PacI, respectively, which are derivatives of pMV361(Kan) [5] engineered to contain a unique *Pac*I restriction site and an apramycin resistance gene in case of pMV361(Apra)-PacI. This resulted in plasmids pMV361(Apra)::*otsB2* and pMV361(Kan)::*otsB2*, respectively, providing constitutive gene expression from the HSP60 promoter. The plasmids were transformed by electroporation into the conditional *M. tuberculosis* c-*otsB2*-tet-on mutant or *M. tuberculosis* wild-type, respectively.

Hidden Markov Models (HMMs) for non-gene-centric comparative analysis of Tn-seq data

A comparative analysis of the differentially essential regions in the Δ*otsA* library grown with- versus without trehalose was performed as follows. Two Hidden Markov Models (HMMs) were used. One was designed to identify regions where there is a clear difference in that the TA sites have insertions in one condition but not the other. The second HMM was desiged to identify regions of quantitative differential essentiality, in that the relative level of insertion counts is significantly lower (but not necessarily zero) in one condition than the other. The advantage of using HMMs to analyze Tn-seq data is that differentially essential regions can be identified in a non-gene-centric way, i.e. not restricted to ORF boundaries.

HMM-1:

A 3-state HMM was implemented in Python to label each TA site as either Essential (ES), Non-essential (NE), or Missing (MI) based on insertion counts at TA sites. The intended interpretation of the MI state is for isolated TA sites where no insertions were observed in the middle of otherwise non-essential regions. The prior probabilities were set at 0.15 for ES, 0.85 for NE, and 0.001 for MI. The transition probability matrix was parameterized as: [[0.001,0.999,0.0],[0.000000001,0.9,0.1],[0.0,0.9999,0.0001]]. The likelihood function for counts in the ES state was given by a geometric distribution, Geom(p=0.9), for the NE was given by a negative Binomial distribution, NegBinom(r=1,p=0.01), and for MI states is 1e-6 for counts > 1 and 1-1e-6 otherwise. The state labels are assigned using the Viterbi algorithm [6].

HMM-2:

Regions with read-counts that were consistently higher in one condition relative to the other were determined using a HMM. The HMM consisted of three states representing regions that had consistently higher read-counts in condition A (S1), consistently higher read-counts in condition B (S3), or read-counts that were more or less evenly distributed (S2). The transition probabilities of the HMM were set so that there was high probability to stay within state

([[0.98,0.01,0.01],[0.01,0.98,0.01],[0.01,0.01,0.98]]). States S1 and S3 had a low prior-probability of being observed (p(S)=0.1 each), as most areas of the genome are not expected to be differentially essential. Observations at states S1 and S3 were modeled using a discrete distribution with the highest mass assigned to the sign of the direction in question: e.g.

P(-1| S1) = 0.700

P(0 | S1) = 0.299

P(1 | S1) = 0.001

The observations at state S2, had a distribution that was closer to uniform as the sign in these regions may change back and forth:

P(-1| S2) = 0.3

P(0 | S2) = 0.4

P(1 | S2) = 0.3

To determine the statistical significance of the difference in counts between the two datasets in each of the type-1 and type-2 segments identified above, a permutation test was performed [5]. Insertion counts at TA sites in each segment were randomly permuted between the datasets 10,000 times to generate a null-distribution for the difference in the sum of the counts between the two datasets, and a p-value for the observed difference was calculated from this. The p-values were then adjusted for multiple comparisions by the Benjamini-Hochberg procedure, and a threshold of adjusted p value <0.05 was applied.

Supplementary References

1. Bardarov S, Bardarov Jr S, Jr., Pavelka Jr MS, Jr., Sambandamurthy V, Larsen M, Tufariello J, et al. Specialized transduction: an efficient method for generating marked and unmarked targeted gene disruptions in Mycobacterium tuberculosis, M. bovis BCG and M. smegmatis. Microbiology. 2002;148(Pt 10):3007-17. Epub 2002/10/09. PubMed PMID: 12368434.

2. Jain P, Hsu T, Arai M, Biermann K, Thaler DS, Nguyen A, et al. Specialized transduction designed for precise high-throughput unmarked deletions in Mycobacterium tuberculosis. MBio. 2014;5(3):e01245-14. doi: 10.1128/mBio.01245-14. PubMed PMID: 24895308; PubMed Central PMCID: PMCPMC4049104.

3. Jain P, Hsu TD, Arai M, Biermann K, Thaler DS, Nguyen A, et al. Specialized Transduction Designed for Precise High-Throughput Unmarked Deletions in Mycobacterium tuberculosis. Mbio. 2014;5(3). doi: ARTN e01245-14

10.1128/mBio.01245-14. PubMed PMID: WOS:000338875900034.

4. Koliwer-Brandl H, Syson K, van de Weerd R, Chandra G, Appelmelk B, Alber M, et al. Metabolic Network for the Biosynthesis of Intra- and Extracellular alpha-Glucans Required for Virulence of Mycobacterium tuberculosis. PLoS Pathog. 2016;12(8):e1005768. doi: 10.1371/journal.ppat.1005768. PubMed PMID: 27513637; PubMed Central PMCID: PMCPMC4981310.

5. Stover CK, de la Cruz VF, Fuerst TR, Burlein JE, Benson LA, Bennett LT, et al. New use of BCG for recombinant vaccines. Nature. 1991;351(6326):456-60. Epub 1991/06/06. doi: 10.1038/351456a0. PubMed PMID: 1904554.

6. Rabiner LR. A tutorial on hidden Markov models and selected applications in speech recognition Proceedings of the IEEE. 1989;77(2):257-86.
