# Supplementary material for: Trehalose-6-Phosphate-Mediated Toxicity Determines Essentiality of OtsB2 in Mycobacterium tuberculosis In Vitro and in Mice
Source: PLoS Pathog. 2016 Dec 9;12(12):e1006043. doi: 10.1371/journal.ppat.1006043 (PMC5148154; doi:10.1371/journal.ppat.1006043)
Supplement: S1 Table — Cells were cultivated in presence of 200 ng/ml (100% growth relative to WT) or 30 ng/ml ATc (ca. 30% residual growth relative to WT) for 7 days. rRNA-depleted samples were analyzed by RNAseq. RPKM, reads per kilobase of transcript per million mapped reads. (PDF) [file ppat.1006043.s011.pdf]

S1 Table. Most abundant transcripts in induced and partially silenced cells of the conditional *M. tuberculosis* c-*otsB2*-tet-on mutant. Cells were cultivated in presence of 200 ng/ml (100% growth relative to WT) or 30 ng/ml ATc (ca. 30% residual growth relative to WT) for 7 days. rRNA-depleted samples were analyzed by RNAseq. RPKM, reads per kilobase of transcript per million mapped reads.

				Average trimmed RPKM	
	Rv ID	Description	Size (bp)	partially silenced	induced
Most abundant in partially silenced cells	RVnc0046	ssr	368	151348.7	301903.6
	RVnc0036a	MTS2823	300	123055.7	856824.6
	Rvnt03	leuT	83	45327.2	152403.1
	Rvns01	rnpB	307	21422.5	66901.6
	Rv1398c	vapB10	258	17890.8	13075.9
	RVnc0006	C8	128	15441.2	122028.7
	RVnc0036	MTS1338	117	13833.1	277857.0
	Rvnt12	aspT	74	13515.8	44517.0
	Rvnt38	metU	74	11107.0	15902.2
	Rv1872c	lldD2	1245	11023.0	7265.6
	Rvnt44	serT	89	9276.9	27669.3
	RVnc0024	mcr7	350	6370.0	5655.6
	Rvnr03	rrf	115	6344.6	11202.3
	RVnc0004	B11	93	5964.1	17216.6
	Rvnt37	alaU	73	5085.8	16496.5
Most abundant in induced cells	RVnc0036a	MTS2823	300	123055.7	856824.6
	RVnc0046	ssr	368	151348.7	301903.6
	RVnc0036	MTS1338	117	13833.1	277857.0
	Rvnt03	leuT	83	45327.2	152403.1
	RVnc0006	C8	128	15441.2	122028.7
	Rvns01	rnpB	307	21422.5	66901.6
	Rvnt12	aspT	74	13515.8	44517.0
	Rvnt44	serT	89	9276.9	27669.3
	RVnc0004	B11	93	5964.1	17216.6
	Rvnt37	alaU	73	5085.8	16496.5
	Rvnt38	metU	74	11107.0	15902.2
	Rv1398c	vapB10	258	17890.8	13075.9
	Rvnr03	rrf	115	6344.6	11202.3
	RVnc0013	mcr11	131	3149.4	7376.8
	Rv1872c	lldD2	1245	11023.0	7265.6
