# Supplementary material for: Trehalose-6-Phosphate-Mediated Toxicity Determines Essentiality of OtsB2 in Mycobacterium tuberculosis In Vitro and in Mice
Source: PLoS Pathog. 2016 Dec 9;12(12):e1006043. doi: 10.1371/journal.ppat.1006043 (PMC5148154; doi:10.1371/journal.ppat.1006043)
Supplement: S2 Table — In order to identify those genes in the M. tuberculosis ΔotsA mutant background whose inactivation cannot be rescued by supplementation with trehalose, i.e. those genes that are essential in context of otsA deletion both in absence and presence of trehalose but non-essential in WT, a saturated transposon mutant pool was generated in the M. tuberculosis ΔotsA mutant background, cultured in the absence of trehalose and subjected to transposon insertion sequencing (Tn-seq). Genes harboring significantly less transposon insertions compared to a transposon mutant library established in M. tuberculosis H37Rv wild-type [27] are shown (p<0.05). Few genes harboring significantly more transposon insertions compared to a transposon mutant library established in M. tuberculosis H37Rv wild-type [27] (i.e. genes appearing less essential in context of otsA gene deletion) are highlighted in grey. (PDF) [file ppat.1006043.s012.pdf]

S2 Table. Differentially essential genes in the *M. tuberculosis* Δ*otsA* mutant compared to wild-type. In order to identify those genes in the *M. tuberculosis* Δ*otsA* mutant background whose inactivation cannot be rescued by supplementation with trehalose, i.e. those genes that are essential in context of *otsA* deletion both in absence and presence of trehalose but non-essential in WT, a saturated transposon mutant pool was generated in the *M. tuberculosis* Δ*otsA* mutant background, cultured in the absence of trehalose and subjected to transposon insertion sequencing (Tn-seq). Genes harboring significantly less transposon insertions compared to a transposon mutant library established in *M. tuberculosis* H37Rv wild-type [27] are shown (p<0.05). Few genes harboring significantly more transposon insertions compared to a transposon mutant library established in *M. tuberculosis* H37Rv wild-type [27] (i.e. genes appearing less essential in context of *otsA* gene deletion) are highlighted in grey.

Rv number	Gene	N of TA sites	TAs Hit	Avg Reads Δ*otsA*	Avg Reads WT [7]	Delta Reads	p-adj
Rv1212c	-	31	22	0	3194.4	3194.4	0
Rv1235	lpqY	30	17	0	1496.1	1496.1	0
Rv1238	sugC	25	9	0	1084.3	1084.3	0
Rv2074	-	6	5	0	1068.1	1068.1	0.0072
Rv0746	PE_PGRS9	15	8	0	862.2	862.2	0.0072
Rv1795	-	22	8	0	1068.4	1068.4	0.0197
Rv1796	mycP5	36	15	6.4	879.9	873.6	0
Rv1236	sugA	19	10	6.4	1109.7	1103.3	0
Rv3512	PE_PGRS56	27	13	6.4	770.4	764	0.0072
Rv1562c	treZ	37	15	6.4	716.4	710.1	0.0072
Rv3664c	dppC	14	6	6.4	285.5	279.1	0.0124
Rv1745c	idi	14	9	6.4	1243.9	1237.5	0.0232
Rv3657c	-	8	6	6.4	434	427.6	0.0249
Rv1262c	-	4	4	6.4	659.5	653.2	0.0415
Rv3590c	PE_PGRS58	21	14	12.7	1815.4	1802.7	0
Rv2236c	cobD	12	6	12.7	1031.3	1018.6	0.0124
Rv1823	-	15	11	12.7	790.7	778	0.0456
Rv3414c	sigD	9	7	19.1	1266.8	1247.7	0.0072
Rv1840c	PE_PGRS34	16	8	19.1	824.1	805	0.0472
Rv2967c	pca	63	19	25.4	1213.1	1187.6	0.0072
Rv1244	lpqZ	8	6	25.4	839.2	813.8	0.0249
Rv1710	-	15	10	25.4	756.3	730.8	0.0429
Rv3388	PE_PGRS52	19	13	31.8	942	910.2	0.0197
Rv2487c	PE_PGRS42	25	15	38.1	2048.8	2010.7	0
Rv0234c	gabD1	23	14	38.1	1095.4	1057.3	0.035
Rv2065	cobH	8	6	38.1	1301.8	1263.6	0.0362
Rv2564	glnQ	14	12	44.5	1396.7	1352.2	0
Rv2159c	-	6	6	50.9	2525.5	2474.6	0.0197
Rv0747	PE_PGRS10	16	11	63.6	2568.7	2505.1	0.0072
Rv3345c	PE_PGRS50	47	25	69.9	2481.5	2411.6	0
Rv1727	-	6	5	69.9	1195.9	1126	0.0249
Rv1564c	treX	49	23	76.3	3404.2	3327.9	0
Rv3563	fadE32	11	11	76.3	1291.9	1215.6	0.0472

Rv0782	ptrBb	45	18	82.6	1406.1	1323.5	0.0197
Rv1991c	-	6	6	82.6	1009.5	926.9	0.0249
Rv2615c	PE_PGRS45	17	9	89	2230.9	2141.9	0
Rv0861c	ercc3	26	16	89	1054.2	965.2	0.0326
Rv1232c	-	11	8	95.4	2506.1	2410.7	0.0232
Rv0279c	PE_PGRS4	19	16	101.7	1886.2	1784.5	0
Rv0012	-	16	12	108.1	2506.8	2398.8	0.0124
Rv2859c	-	17	16	114.4	2384.5	2270	0.0232
Rv3308	pmmB	20	13	114.4	2076.5	1962	0.0276
Rv2800	-	20	17	120.8	2516	2395.2	0
Rv0281	-	14	13	120.8	2232.5	2111.7	0
Rv1283c	oppB	21	14	133.5	2404.3	2270.8	0.0197
Rv0263c	-	16	10	139.9	2938.7	2798.8	0.0072
Rv3787c	-	10	8	146.2	1869.1	1722.9	0.0276
Rv0244c	fadE5	23	18	152.6	1921.6	1769.1	0.0072
Rv0767c	-	14	9	152.6	2695.8	2543.2	0.0232
Rv1910c	-	13	11	158.9	2197.9	2039	0
Rv0092	ctpA	30	22	165.3	3141.6	2976.3	0
Rv0727c	fucA	12	11	171.6	2071.9	1900.2	0.0301
Rv0976c	-	20	17	178	3279.1	3101.1	0.0072
Rv3649	-	33	23	178	1944.7	1766.7	0.035
Rv0889c	citA	12	12	178	2836.6	2658.6	0.0472
Rv1864c	-	10	9	184.4	4396	4211.6	0.0072
Rv1737c	narK2	15	14	190.7	2305.1	2114.4	0.0362
Rv2328	PE23	12	12	190.7	2096.6	1905.9	0.0389
Rv2559c	-	15	13	190.7	3023.3	2832.6	0.0415
Rv3507	PE_PGRS53	41	23	203.4	2805.7	2602.2	0.0362
Rv1180	pks3	25	14	209.8	4939.7	4729.9	0.0389
Rv0613c	-	16	14	235.2	4539.8	4304.6	0
Rv3575c	-	18	15	241.6	1827.3	1585.8	0.0249
Rv3196	-	9	8	241.6	3087.2	2845.7	0.035
Rv0501	galE2	25	18	241.6	2361.3	2119.7	0.0362
Rv0914c	-	16	15	247.9	2206.1	1958.2	0.035
Rv1632c	-	11	11	254.3	3572.3	3318	0.0249
Rv1206	fadD6	37	30	260.6	7149.9	6889.2	0
Rv2681	-	19	15	267	3904.8	3637.8	0
Rv0574c	-	20	15	267	2367.9	2101	0.0072
Rv3220c	-	21	19	273.4	3362.6	3089.3	0.0168
Rv0492c	-	17	14	273.4	3209.5	2936.1	0.0168
Rv3119	moaE1	12	12	273.4	2834.6	2561.2	0.0197
Rv3450c	-	15	13	273.4	4483.1	4209.7	0.0301
Rv2896c	-	20	15	279.7	4269.9	3990.2	0.0168
Rv0630c	recB	34	23	286.1	2913.7	2627.6	0.0472
Rv2066	cobI	22	18	317.8	3836.9	3519.1	0.0124
Rv0210	-	13	9	330.6	2677.7	2347.1	0.0276
Rv2241	aceE	57	18	381.4	0	-381.4	0
Rv2052c	-	21	19	394.1	3413.2	3019.1	0
Rv3312c	-	17	14	400.5	3704.6	3304.1	0.0362
Rv1971	mce3F	20	20	413.2	2890.1	2476.9	0.0498
Rv2860c	glnA4	31	22	432.3	4491.1	4058.8	0.0362
Rv2214c	ephD	31	26	438.6	4148.2	3709.6	0.0072
Rv3731	ligC	18	16	438.6	3293.9	2855.2	0.0124
Rv0877	-	15	13	438.6	4350.2	3911.5	0.0429
Rv3479	-	39	33	445	4758.4	4313.4	0
Rv2458	mmuM	17	14	445	3878.5	3433.5	0.0124
Rv3329	-	21	19	445	4262.3	3817.3	0.0276
Rv0166	fadD5	27	22	451.3	6274.6	5823.3	0
Rv1266c	pknH	29	26	451.3	4400.8	3949.5	0
Rv0443	-	14	12	451.3	4880.1	4428.7	0.0249
Rv0280	PPE3	26	23	457.7	4779.1	4321.4	0
Rv1768	PE_PGRS31	21	20	457.7	6355.1	5897.4	0.0072
Rv2224c	-	29	26	464.1	3149.5	2685.5	0.0362

Rv2329c	narK1	28	24	470.4	5846.8	5376.4	0
Rv2636	-	18	12	476.8	6190.6	5713.8	0.0232
Rv0570	nrdZ	37	28	495.8	3511.3	3015.5	0.0429
Rv1323	fadA4	14	12	514.9	4778.5	4263.6	0.0326
Rv1908c	katG	39	15	534	0	-534	0.0168
Rv0270	fadD2	25	22	540.3	5182.7	4642.4	0.0072
Rv1820	ilvG	20	18	578.5	5790	5211.5	0.0301
Rv1902c	nanT	33	23	616.6	3945.6	3328.9	0.0301
Rv3903c	-	51	33	635.7	4610.3	3974.6	0.0124
Rv2394	ggtB	30	26	654.8	4081.5	3426.8	0.0326
Rv2917	-	25	23	724.7	6088.2	5363.5	0.0429
Rv3822	-	39	26	781.9	6029	5247	0.0197
Rv0191	-	19	16	807.3	8150.2	7342.8	0.0168
Rv0449c	-	23	20	826.4	4807.5	3981.1	0
Rv1770	-	17	14	883.6	6425.9	5542.3	0.0249
Rv1442	bisC	37	33	915.4	7626.5	6711.1	0.0249
Rv1181	pks4	70	50	921.8	14037.9	13116.1	0
Rv3263	-	29	26	921.8	7973.3	7051.6	0.0326
Rv3728	-	32	28	1010.8	5081.7	4070.9	0.0197
Rv2941	fadD28	46	34	1023.5	14827.2	13803.7	0
Rv2000	-	35	30	1023.5	6677.4	5653.9	0.0124
Rv0754	PE_PGRS11	31	22	1029.8	8158.8	7129	0.0197
Rv3296	lhr	55	42	1055.3	10366.7	9311.5	0
Rv3824c	papA1	46	31	1106.1	9284.5	8178.4	0.0276
Rv2209	-	25	19	1195.1	7032.1	5837	0.0197
Rv0386	-	44	36	1246	7395.2	6149.2	0.0249
Rv2115c	-	29	20	1284.1	6.5	-1277.6	0.0124
Rv3059	cyp136	24	22	1411.3	8455.7	7044.5	0.0168
Rv0483	lprQ	29	26	1659.2	12510.2	10851	0
Rv2930	fadD26	40	26	1729.1	19620.4	17891.3	0.0301
Rv0169	mce1A	39	33	1900.7	8807.3	6906.5	0.0429
Rv2931	ppsA	81	62	2027.9	27715.8	25687.9	0
Rv2932	ppsB	71	56	2104.2	19681.6	17577.5	0
Rv0890c	-	48	40	2161.4	10043.3	7881.9	0.0168
Rv2934	ppsD	67	50	2472.9	20525.3	18052.4	0
Rv1836c	-	42	28	3108.6	35.1	-3073.5	0.0072
Rv2690c	-	32	22	3140.4	0.6	-3139.7	0
Rv2933	ppsC	84	66	3324.7	25042.7	21718	0
Rv2935	ppsE	68	58	4303.7	36153.8	31850.1	0
Rv3825c	pks2	116	97	4914	29179.9	24265.9	0
Rv2940c	mas	81	71	8098.8	42390.1	34291.3	0
