# Supplementary material for: Trehalose-6-Phosphate-Mediated Toxicity Determines Essentiality of OtsB2 in Mycobacterium tuberculosis In Vitro and in Mice
Source: PLoS Pathog. 2016 Dec 9;12(12):e1006043. doi: 10.1371/journal.ppat.1006043 (PMC5148154; doi:10.1371/journal.ppat.1006043)
Supplement: S3 Table — Mutants were generated by allelic exchange employing specialized transduction as described in S1 Text. Abbreviations: WT, wild-type; Kanr, kanamycin resistant; Hygr, hygromycin resistant; Apra, apramycin resistant; u, unmarked mutant. (PDF) [file ppat.1006043.s013.pdf]

S3 Table. Strains of *M. tuberculosis* H37Rv used in this study. Mutants were generated by allelic exchange employing specialized transduction as described in S1 Text. Abbreviations: WT, wild-type; Kan^r, kanamycin resistant; Hyg^r, hygromycin resistant; Apra, apramycin resistant; $_u$, unmarked mutant.

Strain	Alternative designation	Relevant characteristics	Source or reference
WT			W.R. Jacobs Jr., AECOM, Bronx, NY
WT pMV361(Kan)::*otsB2*	*otsB2* merodiploid strain	Constitutive *otsB2* expression from the *groEL2* (hsp60) promoter on integrative plasmid pMV361; Kan^r	This study
Δ*otsB2* pMV361(Kan)::*otsB2*		Δ*otsB2*::γδ*res-sacB-hyg*-γδ*res;* constitutive *otsB2* expression from the *groEL2* (hsp60) promoter on integrative plasmid pMV361; Hyg^r, Kan^r	This study
c-*otsB2*-4×*tetO*		Knock-in mutant harboring *hyg*-P*myc1*-4×*tetO* cassette upstream of *otsB2* start codon; Hyg^r	This study
c-*otsB2*-4×*tetO* pMV261(Kan)		Knock-in mutant harboring *hyg*-P*myc1*-4×*tetO* cassette upstream of *otsB2* start codon; empty episomal vector pMV261; Hyg^r, Kan^r	This study
c-*otsB2*-4×*tetO* pMV261(Kan)::*tetR*-G	c-*otsB2*-tet-on	Conditional mutant harboring *hyg*-P*myc1*-4×*tetO* cassette upstream of *otsB2* start codon; constitutive expression of *E. coli* Tn10 *tetR* from the *groEL2* (hsp60) promoter on episomal plasmid pMV261; Hyg^r, Kan^r	This study
c-*otsB2*-4×*tetO* pMV261(Kan)::*tetR*-G pMV361(Apra)::*otsB2*	c-*otsB2*-tet-on pMV361::*otsB2*	Complemented conditional mutant harboring *hyg*-P*myc1*-4×*tetO* cassette upstream of *otsB2* start codon; constitutive expression of *E. coli* Tn10 *tetR* from the *groEL2* (hsp60) promoter on episomal plasmid pMV261; constitutive *otsB2* expression from the *groEL2* (hsp60) promoter on integrative plasmid pMV361; Hyg^r, Kan^r, Apra^r	This study
Δ*otsA*		Δ*otsA*::γδ*res-sacB-hyg*-γδ*res;* Hyg^r	This study
Δ*otsA*$_u$		Δ*otsA*::γδ*res*	This study
Δ*otsA*$_u$ Δ*otsB2*		Δ*otsA*::γδ*res* Δ*otsB2*::γδ*res-sacB-hyg*-γδ*res;* Hyg^r	This study
Δ*otsA*$_u$ c-*otsB2*-4×*tetO*		Δ*otsA*::γδ*res*; Knock-in mutant harboring *hyg*-P*myc1*-4×*tetO* cassette upstream of *otsB2* start codon; Hyg^r	This study
Δ*otsA*$_u$ c-*otsB2*-4×*tetO* pMV261(Kan)::*tetR*-G	Δ*otsA*$_u$ c-*otsB2*-tet-on	Δ*otsA*::γδ*res*; Conditional mutant harboring *hyg*-P*myc1*-4×*tetO* cassette upstream of *otsB2* start codon; constitutive expression of *E. coli* Tn10 *tetR* from the *groEL2* (hsp60) promoter on episomal plasmid pMV261; Hyg^r, Kan^r	This study
Δ*panCD*$_u$		Δ*panCD*::γδ*res*	W.R. Jacobs Jr., AECOM, Bronx, NY
Δ*panCD*$_u$ c-*otsB2*-4×*tetO*		Knock-in mutant harboring *hyg*-P*myc1*-4×*tetO* cassette upstream of *otsB2* start codon; Δ*panCD*::γδ*res*; Hyg^r	This study
Δ*panCD*$_u$ c-*otsB2*-4×*tetO* pMV261(Kan)::*tetR*-G::*panCD*	Δ*panCD*$_u$ c-*otsB2*-tet-on	Conditional mutant harboring *hyg*-P*myc1*-4×*tetO* cassette upstream of *otsB2* start codon; Δ*panCD*::γδ*res*; constitutive expression of *E. coli* Tn10 *tetR* and *M. tuberculosis* H37Rv *panCD* as an artificial tricistronic operon from the *groEL2* (hsp60) promoter on episomal plasmid pMV261; Hyg^r, Kan^r	This study
