# Supplementary material for: Trehalose-6-Phosphate-Mediated Toxicity Determines Essentiality of OtsB2 in Mycobacterium tuberculosis In Vitro and in Mice
Source: PLoS Pathog. 2016 Dec 9;12(12):e1006043. doi: 10.1371/journal.ppat.1006043 (PMC5148154; doi:10.1371/journal.ppat.1006043)
Supplement: S4 Table — Resulting phages listed here were used for generation of gene deletion or knock-in mutants of M. tuberculosis H37Rv listed in S3 Table by specialized transduction as described in S1 Text. (PDF) [file ppat.1006043.s014.pdf]

S4 Table. Oligonucleotides used for generation of allelic exchange substrates. Resulting phages listed here were used for generation of gene deletion or knock-in mutants of *M. tuberculosis* H37Rv listed in S3 Table by specialized transduction as described in S1 Text.

	**Upstream flanking sequence**			**Downstream flanking sequence**			**Resulting phage**
	**5` primer**	**3` primer**	**Restriction site**	**5` primer**	**3` primer**	**Restriction site**	
ΔotsA	5` TTTTTCCATAAATTGGGCGTGGCTG-ACCCAAGAACT 3`	5` TTTTTCCATTTCTTGGTCGATTGGC-TACCACCACGA 3`	*Van*91I	5` TTTTTGCATAGATTGCCTGGGCACA-GTCGTTTCTCG 3`	5` TTTTGCATCTTTTGCCACCTGGAAG-GTCCACAGCA 3`	*BstAP*I	phRv3490S
ΔotsB2	5` TTTTTGCATAAATTGCCCCTATCTC-CCCGTCGAGTACGAC 3`	5` TTTTTGCATTTCTTGCGAGTTGCCG-GACCAGTTCCTGGG 3`	*BstAP*I	5` TTTTTCCATAGATTGGGCACTGTTT-GCGCTGGACAGTCC 3`	5` TTTTTCCATCTTTTGGCATGCGGGT-GTTCGGAAACTCCTTG- 3`	*Van*91I	phRv3372S
c-otsB2	5` TTTTTGCATAAATTGCCATGTCGGC-GGCCCACCTGCTG 3`	5` TTTTTGCATTTCTTGCGACCCACCCT-CTCACGGGGGTC 3`	*Van*91I	5` TTTTTGCATAGATTGCATGCGCAAG-TTGGGCCCGGTC 3`	5` TTTTTGCATCTTTTGCTTCTGCAGT-GCCTCCAAGGC 3`	*Van*91I	phcRv3372-4xtetO
