# Supplementary material for: Trehalose-6-Phosphate-Mediated Toxicity Determines Essentiality of OtsB2 in Mycobacterium tuberculosis In Vitro and in Mice
Source: PLoS Pathog. 2016 Dec 9;12(12):e1006043. doi: 10.1371/journal.ppat.1006043 (PMC5148154; doi:10.1371/journal.ppat.1006043)
Supplement: S5 Table — (PDF) [file ppat.1006043.s015.pdf]

S5 Table. Oligonucleotides used for qRT-PCR of *M. tuberculosis* transcripts.

Gene	Forward primer	Reverse primer
vapB43 (*Rv2871*)	5` ACCGCGAGGTGAAAGCAAAGG 3`	5` AAGTGCGGCGTTGGACGATAG 3`
vapB7 (*Rv0662*)	5` AATGGCCTCACGCACGACGGTG 3`	5` TACGCGTGCCTATCGACGCTAC 3`
Rv1258c	5` ACCGATGGCCGGGCCGACAATAAAG 3`	5` AACGTGCTGGTGCTGGCCGTATTGG 3`
Rv1655 (argD)	5` CGACGTGGACGGCAGAACCTATATC 3`	5` GCCGGAGTTGCAGAAGAACACTCG 3`
Rvnc0036a (MTS2823)	5` AAGGCTCGATCCAGAAGAGAAGG 3`	5` CAACACGGTTCTCGGTTACCAAG 3`
Rv3371 (*otsB2*)	5` GAACTGGTCCGGCAACTCCAGGAAG 3`	5` TGCGGTCGACACCGATGATCAGC 3`
16S rRNA	5` GAGTGGCGAACGGGTGAGTAAC 3`	5` GGAGTCTGGGCCGTATCTCAGTC 3`
